# Deletion of MtrA Inhibits Cellular Development of *Streptomyces coelicolor* and Alters Expression of Developmental Regulatory Genes

**DOI:** 10.3389/fmicb.2017.02013

**Published:** 2017-10-16

**Authors:** Peipei Zhang, Lili Wu, Yanping Zhu, Meng Liu, Yemin Wang, Guangxiang Cao, Xiu-Lan Chen, Meifeng Tao, Xiuhua Pang

**Affiliations:** ^1^The State Key Laboratory of Microbial Technology, School of Life Sciences, Shandong University, Jinan, China; ^2^The State Key Laboratory of Microbial Metabolism, Shanghai Jiao Tong University, Shanghai, China; ^3^Shandong Medicinal Biotechnology Center, Shandong Academy of Medical Sciences, Jinan, China

**Keywords:** *Streptomyces*, MtrA, development, *bld*, *whi*

## Abstract

The developmental life cycle of *Streptomyces* species includes aerial hyphae formation and spore maturation, two distinct developmental processes that are controlled, respectively, by two families of developmental regulatory genes, *bld* and *whi*. In this study, we show that the response regulator MtrA (SCO3013) is critical for normal development of aerial hyphae in *S. coelicolor* and related species. Δ*mtrA,* a deletion mutant of the response regulator gene *mtrA*, exhibited the bald phenotype typical of *bld* mutants defective in aerial mycelium formation, with formation either much delayed or absent depending on the culture medium. Transcriptional analysis indicated that MtrA activates multiple genes involved in formation of aerial mycelium, including *chp*, *rdl*, and *ram* genes, as well as developmental regulatory genes of the *bld* and *whi* families. However, the major regulatory gene *bldD* showed enhanced expression in Δ*mtrA*, suggesting it is repressed by MtrA. electrophoretic mobility shift assays indicated that MtrA binds upstream of several genes with altered expression in Δ*mtrA*, including *bldD* and *whiI*, and sequences similar to the consensus binding sequence for MtrA of another actinomycete, *Mycobacterium tuberculosis*, were found in the bound sites. A loosely conserved recognition sequence containing two short, direct repeats was identified for MtrA of *S. coelicolor* and was validated using mutational analysis. MtrA homologs are widely distributed among *Streptomyces* species, and as with *S. coelicolor,* deletion of the *mtrA* homologs *sve_2757* from *S. venezuelae* and *sli_3357* from *S. lividans* resulted in conditional bald morphology. Our study suggests a critical and conserved role for MtrA in *Streptomyces* development.

## Introduction

Streptomycetes are multicellular, filamentous, Gram-positive bacteria that possess two extraordinary traits rarely seen in other prokaryotes. First, *Streptomyces* produce a great variety of secondary metabolites that account for more than half of clinically important agents, including antimicrobial and anti-tumor medicines (Hopwood, 2007). Secondly, *Streptomyces* exhibit a complex developmental life cycle, with the formation of substrate (vegetative) mycelium, aerial mycelium, and spores at different growth stages during development (Flardh and Buttner, 2009; Chater, 2011; McCormick and Flardh, 2011).

Development processes of *Streptomyces* have been studied primarily in the model strain *S. coelicolor* (Flardh and Buttner, 2009; Chater, 2011; McCormick and Flardh, 2011). To grow upward into the air, the aerial hyphae need to be coated with a hydrophobic sheath, which is composed mainly of two types of hydrophobic proteins, chaplins and rodlins, whose expression is developmentally regulated (Claessen et al., 2003; Elliot et al., 2003; Talbot, 2003). The eight chaplin proteins of *S. coelicolor,* ChpA-H, have been categorized into two types according to size and structure. ChpA-ChpC are large proteins with two chaplin domains and a C-terminal sorting signal, whereas ChpD-ChpH are relatively small proteins with only one chaplin domain (Claessen et al., 2003; Elliot et al., 2003). The long chaplin proteins become covalently attached to the cell wall peptidoglycan of the aerial hyphae and spores by sortase enzymes and are resistant to separation from the cell wall (Claessen et al., 2003; Duong et al., 2012). There appears to be some redundancy among the chaplin proteins as only the simultaneous deletion of multiple *chp* genes attenuated aerial hyphae formation and reduced attachment to substrate surfaces (Claessen et al., 2004; de Jong et al., 2009). Like chaplins, rodlins are structural components of aerial mycelium in *S. coelicolor* (Claessen et al., 2002). Rodlin proteins are detected only in cultures forming aerial hyphae, and although disruption of *rdlA* and *rdlB* does not affect the formation of aerial structure, it does affect the formation of the rodlet layer (Claessen et al., 2002, 2004). Studies indicate that rodlins and chaplins interact to form the rodlet layer and that the rodlins weave chaplins into paired rodlets displaying a characteristic basketwork-like appearance on the surface of *S. coelicolor* spores (Wildermuth, 1970; Wildermuth et al., 1971; Claessen et al., 2002, 2004).

For differentiation into aerial hyphae, the substrate mycelium of *S. coelicolor* also requires SapB, a small, hydrophobic, morphogenetic peptide, to reduce surface tension at the colony interface on rich solid media such as R2YE (Willey et al., 1991, 1993, 2006; Tillotson et al., 1998; Wosten and Willey, 2000). Production of SapB requires the *ram* (rapid aerial mycelium formation) gene cluster *ramCSAB* (Willey et al., 1991; Ma and Kendall, 1994; Kodani et al., 2004). The regulatory gene *ramR,* immediately adjacent to *ramB,* plays a pivotal role in the regulation of SapB production, as RamR activates the *ramC* operon by binding directly to sequences upstream (Nguyen et al., 2002; O’Connor et al., 2002). Evidence suggests that SapB production is also influenced by the membrane localization of the precursor peptide RamS (Gaskell et al., 2012). It was proposed that both SapB and the chaplins are essential for normal aerial formation on rich media such as R2YE, whereas chaplins alone drive aerial morphogenesis on complex medium such as solid MS medium (Claessen et al., 2004; Capstick et al., 2007).

The regulation of development in streptomycetes is only partially understood, although *bld* and *whi* genes are known to be involved (Flardh and Buttner, 2009; Chater, 2011; McCormick and Flardh, 2011). *bld* mutations block the formation of aerial mycelium, resulting in a ‘bald’ appearance, and *whi* mutations block steps in the conversion of aerial mycelia to mature, gray spores, and hence *whi* mutants appear ‘white’ (Flardh and Buttner, 2009; Chater, 2011; McCormick and Flardh, 2011). A developmental regulatory cascade involving most *bld* and *whi* genes was established after years of study, with BldD apparently at the top of the regulatory cascade (Den Hengst et al., 2010; Bush et al., 2016). BldD is an auto-regulatory, DNA-binding protein (Elliot et al., 1998; Elliot and Leskiw, 1999), and its targets includes *bldA*, *bldC*, *bldH*, *bldM*, *bldN*, *whiB*, and *whiG* (Elliot et al., 2001; Den Hengst et al., 2010; Bush et al., 2016). To regulate its target genes, BldD needs to form a homodimer, a process that depends on c-di-GMP (Tschowri et al., 2014), and therefore BldD activity is essentially influenced by the level of this signal molecule (Tschowri et al., 2014). Recent research also provides evidence that collaboration between *bld* and *whi* factors (BldM and WhiI) or among *whi* factors (WhiA and WhiB) is required to control the key stages in *Streptomyces* development (Bush et al., 2013, 2016; Al-Bassam et al., 2014).

The genome of *S. coelicolor* encodes 67 paired two-component signal transduction systems (TCSs), 13 orphan response regulators, and 17 orphan sensor kinases (Bentley et al., 2002; Hutchings et al., 2004). Some TCSs have been characterized as regulators of primary or secondary metabolism (Hutchings et al., 2004); for example, PhoPR, the most studied TCS in *S. coelicolor*, is implicated in both phosphate metabolism and antibiotic production (Fink et al., 2002; Sola-Landa et al., 2003, 2005), whereas others are involved in secondary metabolism. Some TCSs have been implicated in development, such as BldM (Molle and Buttner, 2000), RamR (Keijser et al., 2002; Nguyen et al., 2002; O’Connor et al., 2002), and WhiI (Ainsa et al., 1999; Al-Bassam et al., 2014). However, the function of most of these signal systems and their roles in developmental regulation have not been fully explored.

SCO3012/3013 is one of the uncharacterized TCSs in *S. coelicolor,* and SCO3013 is annotated as the response regulator of this system. SCO3013 has 75% amino acid identify to MtrA (MtrA_MTB_) of *Mycobacterium tuberculosis*, which is an essential gene in that species (Zahrt and Deretic, 2000, 2001), and 69% amino acid identity to MtrA (MtrA_CGL_) of *Corynebacterium glutamicum*, which is implicated in the regulation of cell wall metabolism and osmoprotection (Moker et al., 2004); therefore, SCO3013 was also named MtrA (Hoskisson and Hutchings, 2006). In *S. coelicolor, mtrA* (*sco3013*), its cognate sensor kinase gene *mtrB* (*sco3012*), and the flanking gene *lpqB* (*sco3011*), which encodes a putative lipoprotein, form an operon that is conserved in other actinobacteria, with MtrA displaying the highest sequence conservation of the three genes (Hoskisson and Hutchings, 2006). The critical biological role of MtrA in other gram-positive actinomycetes suggests that MtrA may have an important role in *Streptomyces*. Here, we report that MtrA is critical for development and that this role is conserved in streptomycetes.

## Materials and Methods

### Bacterial Strains and Culture Conditions

Bacterial strains and plasmids used in this study are listed in Supplementary Table S1. The wild-type *S. coelicolor* and *S. lividans* strains and their derivatives were grown at 30°C on mannitol soya (MS) flour agar for spore production and conjugal transfer; on TSBY broth medium (3% tryptone soya broth, 10.3% sucrose, 0.5% yeast extract, final pH of 7.2) for mycelium preparation (Pospiech and Neumann, 1995); and on complex or rich medium (Kieser et al., 2000; Ou et al., 2009) media to observe morphological phenotypes. Various *Escherichia coli* strains were cultivated in Luria-Bertani (LB) liquid medium and used as indicated in Supplementary Table S1. Ampicillin (100 μg ml^-1^), apramycin (50 μg ml^-1^), thiostrepton (20 μg ml^-1^), kanamycin (25 μg ml^-1^), hygromycin (50 μg ml^-1^), chloramphenicol (25 μg ml^-1^), and nalidixic acid (25 μg ml^-1^) were added, as appropriate, to growth medium for selection of either *E. coli* or *Streptomyces* transformants.

### Construction of the *S. coelicolor* M145 Genomic Library and Southern Blotting

Chromosomal DNA was isolated from *S. coelicolor* M145 mycelia grown in 50 mL TSBY broth containing 0.5% glycine, as described (Pospiech and Neumann, 1995). The genomic DNA was partially digested with *Mbo*I to yield DNA fragments of 35–45 kb, which were then ligated into the vector SuperCos I cosmid that had been previously linearized with *Xba*I, dephosphorylated, and digested with *Bam*HI. The resulting ligation mixture was packaged into λ phage, followed by phage transfection into *E. coli* XL1-Blue MR using the MaxPlax Lambda Packaging Extracts Kit (Epicentre), according to the manufacturer’s instructions. Ampicillin-resistant colonies were grown in LB medium supplemented with ampicillin, and then stored in 25% glycerol at -80°C.

Primers 3013-hybrid-foward/reverse (Supplementary Table S2) that encompass the *mtrA* coding sequence were used to generate a 1065-bp DNA fragment, which was purified and labeled using reagents supplied in the DIG High Prime DNA Labeling and Detection Starter Kit II (Roche); the labeled fragment was then used to probe a M145 cosmid library that had been transferred to nylon membranes using standard hybridization protocols (Sambrook and Russell, 2001). Finally, after addition of detection buffer, the membrane was exposed to X-ray film for 20 min to detect the chemiluminescent signal. Cosmid 19E7 generated a positive signal with the probe, and its insert was determined using inward-sequencing with primers specific to the cloning sites of the vector.

### Deletion of *mtrA* from *S. coelicolor* M145

A PCR-targeting system was used to delete *mtrA* from *S. coelicolor* essentially as described (Gust et al., 2003). Briefly, a disruption cassette was generated by PCR using pIJ773 as template with primer pair *mtrA*-Target-F/R (Supplementary Table S2), and then electro-transformed into *E. coli* BW25113, which contains pIJ790 that provides the λ-Red system to enhance recombination, and cosmid 19E7, which contains the *mtrA* gene, to generate the *mtrA*-deleted cosmid Δ*mtrA*::*aac(3)IV*. Deletion of *mtrA* was verified by both PCR using primer pair Δ*mtrA*-Confirm-F/R (Supplementary Table S2) and by restriction enzyme digestion analysis. Cosmid Δ*mtrA*::*aac(3)IV* was then introduced into the non-methylating *E. coli* strain 12567/pUZ8002 before transferring into *S. coelicolor*M145 by conjugation. After several rounds of selection on MS agar containing apramycin, the deletion of *mtrA* from the apramycin-resistant exconjugant Δ*mtrA* was confirmed by PCR using the primer pair Δ*mtrA*-Confirm-F/R.

Owing to the high sequence identity (99–100%) between *sli_3357* of *S. lividans* and *mtrA* of *S. coelicolor* and the strong sequence similarity between their flanking sequences, the same mutated cosmids were used to generate mutant Δ*sli_3357* in *S. lividans* 1326.

### Deletion of *sven_2756* from *S. venezuelae*

To mutate *sven_2756* in *S. venezuelae*, a 2108-bp flanking sequence at the left side of the gene was used as the left arm for homologous recombination and was amplified using primer pair SVEN2756-L Forward/Reverse (Supplementary Table S2), which carry an *Xba*I and *Spe*I site, respectively. The 2108-bp DNA fragment was then cloned into pEasy-Blunt to generate p-LArm. A 2071-bp flanking sequence at the right side of the gene was used as the right arm for homologous recombination and was amplified using primer pair SVEN2756-R Forward/Reverse (Supplementary Table S2), which carry an *Spe*I site and a *Hind*III site, respectively; this fragment was also inserted into pEasy-Blunt to generate p-RArm. A third fragment, 1269-bp in length and containing a kanamycin cassette, was amplified using primer pair Kana-Forward/Reverse and cloned into pMD18-T. After sequence verification, the right arm was released from p-RArm by *Spe*I and *Hind*III digestion, ligated with p-LArm pretreated with *Hind*III and *Spe*I, and the resulting plasmid pL-R was verified by digestion with *Hind*III and *Xba*I. Next, the kanamycin cassette was removed by *Spe*I digestion, purified, and ligated with pL-R pretreated with *Spe*I (to separate the left and the right arm); the resulting plasmid pL-K-R was verified by digestion with *Hind*III and *Xba*I. Then, the fragment containing the left and right arms and the resistance cassette was released by *Hind*III and *Xba*I digestion, purified, and inserted into pJTU1278 pretreated with *Hind*III and *Xba*I. The resulting plasmid was verified by restriction analysis and was designated pMu-2756.

pMu-2756 was introduced into the non-methylating *E. coli* strain 12567/pUZ8002, and the transformants were used as donor strains in conjugation. The protocol for conjugation and selection of mutants was essentially as described for *S. coelicolor* M145, except that R2-S medium was used for conjugation as described (Bibb et al., 2012). After several rounds of selection on R2S agar containing kanamycin, the deletion of *sven_2756* from the exconjugant was confirmed by PCR using the primer pair SVEN2756-C Forward/Reverse (Supplementary Table S2).

### Genetic Complementation

To generate a complemented strain for Δ*mtrA*, the upstream intergenic region and coding sequence of *mtrA* or *mtrA* and *mtrB* were amplified by PCR, using corresponding primer pairs (Supplementary Table S2). The amplicon was purified, inserted into pCR-Blunt, and following sequencing verification, the insert was excised and ligated with the pre-cut integrating plasmid pMS82 (Gregory et al., 2003), which confers hygromycin resistance, to generate pCom-3013_SCO_ and pCom-3012/13_SCO_, respectively. Also based onpMS82, pCom-2757_SV EN_ was constructed, which contains the upstream intergenic sequence and the coding sequence of *sven_2756* of *S. venezuelae*. All plasmid constructs were verified by sequencing and were introduced into mutant strains by conjugation to generate the complemented strains (Supplementary Table S1). Exconjugants with resistance to hygromycin were screened and confirmed by PCR analysis.

### RNA Isolation, Reverse Transcription-PCR (RT-PCR), and Real-time PCR

To extract RNA, wild-type and mutant strains of *Streptomyces* were grown at 30°C on YBP solid medium covered with plastic cellophane, and the mycelia were collected at various times, ground in liquid nitrogen, and then dispensed into Rezol reagent (SBSBIO). Crude RNA samples were treated twice with ‘Turbo DNA-free’ DNase reagents (Ambion) to remove chromosomal DNA, and reverse transcription was carried out, as described (Zhang et al., 2014). The SYBR *Premix Ex Taq* (TaKaRa) was used under recommended conditions on a Roche LightCycler480 thermal cycler to determine the melting curve of PCR products and their specificity, and for real-time PCR assays. Relative quantities of cDNA were normalized for the *hrdB* gene, which encodes the major sigma factor of *Streptomyces*. Sequences of primers used in RNA analysis are listed in Supplementary Table S2.

### RNA-Seq and Data Analysis

For RNA-Seq analysis, the total RNA of *S. coelicolor* M145 and Δ*mtrA* was extracted from cultures incubated at 30°C for 72 h on solid YBP medium covered with cellophane and were then treated with RNase-free DNase I (Invitrogen) twice according to the recommended protocols. Two sets of RNA for both strains were prepared separately. The integrity of total RNA was determined using a Thermo NanoDrop, and the RNA Integrity Number value of each sample met the standard required for preparing a cDNA library. The cDNA libraries were prepared according to the manufacturer’s instructions (Illumina). Briefly, rRNA in 1 μg total RNA was depleted using Ribo-zero rRNA Removal solution (Illumina), leaving only mRNA, and the mRNA was then fragmented into small pieces in fragment mix at elevated temperature to avoid priming bias when synthesizing cDNA. Using random primers and Super Script II (Invitrogen), the small mRNA fragments were then converted into double-stranded cDNA by first- and second-strand cDNA synthesis, followed by end-repair and 3′-adenylation to produce cDNA fragments with a single ‘A’ base overhang at their 3′-ends; adapters were then ligated to the ends of the cDNA fragments. Fifteen rounds of PCR amplification were performed to enrich the adapter-modified cDNA library using primers complementary to the ends of the adapters, and PCR products were purified using Ampure XP beads (Agencourt). The concentration of the cDNA library was determined with a Taqman probe using ABI StepOnePlus Real-time PCR system, and the size range of the library insert was determined with Agilent DNA 1000 Reagents using the Agilent 2100 Bioanalyzer. The cDNA library products were denatured into single strands with the addition of NaOH, diluted to optimal concentration, and loaded into a FlowCell to hybridize with the adapter fixed on the FlowCell. Bridge-PCR amplification was performed with TruSeq PE Cluster Kit V3-cBot-HS (Illumina), and the FlowCell samples were sequenced using HiSeq2000 (Illumina).

The expression level of each gene was normalized by the number of reads per kilobase of transcriptome per million mapped reads (RPKM). The cut-off value for determining gene transcriptional activity was determined based on a 95% confidence interval for all RPKM values of each gene.

### Construction of an *mtrA* Expression Plasmid and Purification of Protein MtrA

The *mtrA* coding sequence was amplified using primers *mtrA*-Exp-F (with an *Nde*I adaptor) and *mtrA*-Exp-R (with a *Hind*III adaptor) (Supplementary Table S2), and the PCR product was purified by agarose gel electrophoresis and inserted into pMD18-T (Takara). After sequence verification, the inserts were excised by *Nde*I and *Hind*III digestion, gel-purified, and ligated into *Nde*I/*Hind*III-cut pET28a (Invitrogen) to generate pEX-*mtrA*, which was used to transform *E. coli* Rosetta(DE3)pLysS (Novagen). Expression of MtrA was induced by the addition of isopropyl β-D-1-thiogalactopyranoside (1.0 mM) when the cell density was around 0.6 (at OD_600__nm_), with incubation for 4–5 h at 30°C. Cell lysates were prepared by sonication in binding buffer (50 mM NaH_2_PO_4_, 250 mM NaCl, 20 mM imidazole, pH 8.0), and the His-tagged MtrA was purified using Ni-NTA-Sefinose Column (Sangon.), using washing buffer (50 mM NaH_2_PO_4_, 250 mM NaCl, 40 mM imidazole, pH 8.0) and then elution buffer (50 mM NaH_2_PO_4_, 250 mM NaCl, 250 mM imidazole, pH 8.0). Purified protein was then dialyzed in dialysis cassettes (10,000 MWCO, Thermo Scientific) in a dialyzing buffer (50 mM NaH_2_PO_4_, 50 mM NaCl, pH 8.0) before concentrating with centrifugal filters (10,000 MWCO, Millipore). Protein concentration was determined using the bicinchoninic acid assay (Pierce).

### Scanning Electronic Microscopy (SEM)

Briefly, spores of *S. coelicolor* M145 and its derivative strains were inoculated onto MS agar medium; for *S. venezuelae* strains, YBP agar medium was used instead. A sterile coverslip was inserted into the agar at an angle to allow the culture to overgrow its surface. After 5 days of incubation at 30°C, the coverslip was removed, fixed with fresh 2% glutaraldehyde (pH 7.2) for 2 h at 30°C, and washed three times with 0.1 M PBS buffer (pH 7.0) before treating with 1% osmic acid. The coverslips were then dehydrated by soaking in a series of ethanol gradients, dried in a Leica EM CPD300 Critical Point Dryer, coated with gold in a Cressinton Sputter Coater 108, and examined with a scanning electron microscope (FEI Quanta250 FEG, United States).

### Electrophoretic Mobility Shift Assays (EMSAs)

DNA probes containing the upstream region of selected genes were generated by PCR amplification using specific primer pairs as listed (Supplementary Table S2). For short probes containing putative MtrA binding sequence, complementary oligonucleotides were annealed. The DNA probes were labeled with biotin-11-UTP using the Biotin 3′ End DNA Labeling Kit (Thermo Scientific), according to the manufacturer’s instruction, and 50 fmol of labeled probes were mixed with differing amounts of purified MtrA in binding buffer (20 mM Tris-HCI, 2 mM EDTA, 20 mM KCI, 0.5 mM DTT, 4% Ficoll-400, pH 8.0). For an unspecific competitor, 2 μg of poly (dI-dC) was included in the reactions (20 μl total reaction volume). The DNA and protein mixes were incubated at room temperature for 15 min, and then were separated on 8% non-denaturing polyacrylamide gels. After gel separation, the DNA was transferred to and fixed on nylon membrane, and then blocked, washed, and processed before signal detection using the ECL Western Blotting Analysis System kit (GE Healthcare) and exposure to X-film.

### DNaseI Foot-Printing Assay

The DNA probes used in electrophoretic mobility shift assays (EMSA) were cloned into pMD18-T to generate plasmid templates. For preparation of fluorescent FAM-labeled probes, the promoter region of each gene was PCR amplified with Dpx DNA polymerase (TOLO Biotech) from the plasmid template using primers M13F-47 (FAM) and M13R-48. The FAM-labeled probes were purified by the Wizard^®^ SV Gel and PCR Clean-Up System (Promega) and were quantified with NanoDrop 2000C (Thermo). DNase I footprinting assays were performed essentially as described (Wang et al., 2012). For each assay, 400 ng of probe was incubated with different amounts of purified MtrA in a total volume of 40 μl, which also included poly (dI-dC) as the unspecific competitor. After incubation for 30 min at 25°C, 10 μl of solution containing approximately 0.015 units DNase I and 100 nmol freshly prepared CaCl_2_ was added, followed by further incubation for 1 min at 25°C. The reaction was stopped by adding 140 μl DNase I stop solution (200 mM unbuffered sodium acetate, 30 mM EDTA, and 0.15% SDS). Samples were extracted with phenol/chloroform, precipitated with ethanol, and the pellets were dissolved in 30 μl MiniQ water. Preparation of the DNA ladder, electrophoresis, and data analysis were performed as described previously (Wang et al., 2012; Cao et al., 2015), except that the GeneScan-LIZ500 size standard (Applied Biosystems) was used.

### Identification of an MtrA Consensus Recognition Sequence

The sequences protected or shifted by MtrA in the regions upstream of *sco1189*, *sco1489*, *sco1568*, *sco2136*, *sco2210*, *sco3485*, *sco3561*, *sco3863*, *sco5583*, *sco6029*, *sco7434*, and *sco7458* were used as input for the *MEME* software tool (Bailey and Elkan, 1994) for the MtrA_SCO_ motif search.

## Results

### Disruption of *mtrA* Leads to a Typical Bald Phenotype in *S. coelicolor*

**FIGURE 1:**
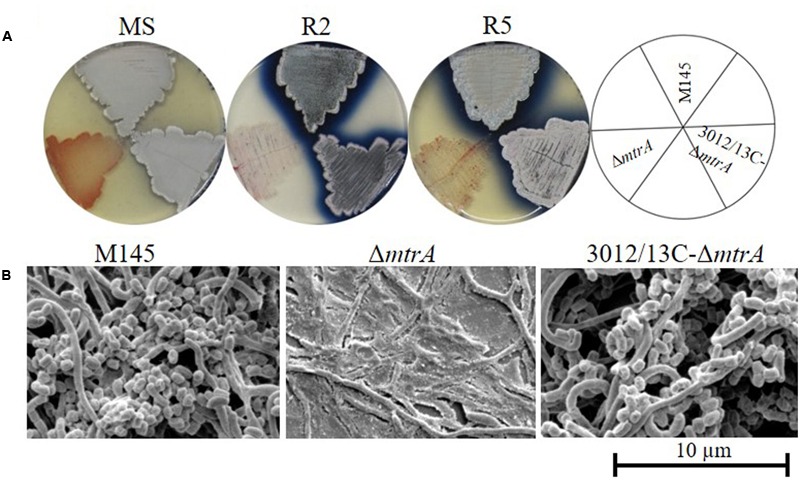
Requirement of *mtrA* for the formation of aerial hyphae in *S. coelicolor.* **(A)** Phenotypes of the *S. coelicolor* wild-type strain M145, the *mtrA* deletion mutant Δ*mtrA*, and the *mtrA*-complemented strain C-Δ*mtrA* grown at 30°C on solid MS (7 days), R2 (6 days), and R5 (6 days) media. **(B)** SEM images of M145, Δ*mtrA*, and C-Δ*mtrA* after growth on MS agar for 5 days. Images reveal the classic ‘bald’ phenotype for the deletion mutant and normal aerial hyphae for the wild-type and complemented strains. The scale bar is 10 μm.

We screened a transposon library of *S. coelicolor* M145 for mutants with altered production of pigmented antibiotics (Xu et al., 2017), discovering four mutants with the bald phenotype typical of strains lacking aerial hyphae; these mutants contained transposons within the coding sequence of *mtrA*, suggesting a role for MtrA in developmental control. To verify the effects of MtrA on *Streptomyces* morphology, we generated the deletion mutant Δ*mtrA*, which lacks the entire *mtrA* coding sequence, in strain *S. coelicolor* M145. Δ*mtrA* exhibits significant morphological differences from strain M145 when grown on various media (**Figure 1A** and data not shown). On MS medium, formation of aerial mycelia was severely delayed in Δ*mtrA*, with none observed even after 8 days of growth (**Figure 1A**), in sharp contrast to M145, which began formation of aerial mycelia as early as 48 h under the test conditions. However, after prolonged incubation on MS medium (∼10 days), Δ*mtrA* was capable of developing aerial mycelia, with mature spores forming after approximately 2 weeks. Notably, on R2, R5, R2YE (Kieser et al., 2000) and YBP (Ou et al., 2009), Δ*mtrA* maintained a ‘permanent’ bald phenotype, with no aerial mycelia observed even after weeks of growth, whereas wild-type M145 began forming aerial mycelia at 48 h under the same conditions. Overall, Δ*mtrA* displayed a striking morphological change, i.e., a bald phenotype that was conditional upon the growth medium, a very similar phenotype to that reported for SapB- and chaplin-deficient mutants and *bld* mutants (Capstick et al., 2007; Chater, 2011). We further observed that Δ*mtrA* cultures appeared very ‘wet’ on rich media and that water droplets spread easily over the Δ*mtrA* culture surface, suggesting that, compared to the wild-type strain, this mutant has a much more hydrophilic nature.

To confirm that the profound phenotypic changes in Δ*mtrA* were directly due to mutation of *mtrA*, we introduced plasmid pCom-3012/13_SCO_, which contains the coding sequence of *sco3012* (*mtrB*) and *sco3013* (*mtrA*) and the upstream intergenic sequence, into Δ*mtrA* to generate the complemented strain 3012/13C-Δ*mtrA*; this complementation restored the growth of aerial mycelium and spore formation to a level comparable to that of wild-type M145 (**Figure 1A**), confirming a critical role for *mtrA* in developmental control in *S. coelicolor*. However, when pCom-3013_SCO_, which contains the coding sequence of *sco3013* (*mtrA*) and the upstream intergenic sequence, was introduced into Δ*mtrA*, the phenotype was only partially complemented in strain 3013C-Δ*mtrA* under most conditions tested. As there is only one nucleotide space between the putative start codon of *mtrB* and the annotated stop codon of *mtrA*, and as the entire coding sequence of *mtrA,* except for the start and stop codons, was replaced by an apramycin cassette, translation of MtrB may be impaired in Δ*mtrA*. However, the transcription of *mtrB* did not appear to be affected by the *mtrA* mutation, as inferred from RNA-Seq data in which *mtrA* was the most down-regulated gene due to the absence of the coding sequence, whereas no obvious change was detected for *mtrB*. It is possible, though, that the inefficient complementation of Δ*mtrA* by *mtrA* alone (pCom-3013_SCO_) compared to *mtrAB* (pCom-3012/13_SCO_) under some conditions, such as growth on R2 and R5 media, might be due to a polar effect of the *mtrA* mutation. However, the fact that under other conditions, such as growth on MS medium, *mtrA* complements the deletion nearly as well as *mtrAB* argues that any such polar effect is not strong. Nevertheless, the results from this and other studies indicate that MprB is required for maintaining the full activity of MprA (Som et al., 2017), consistent with MprA and MprB composing a two-component system.

### Morphological Analysis by Scanning Electron Microscopy (SEM)

In other *Streptomyces* mutants, the bald phenotype is associated with the absence of aerial mycelia. Therefore, the morphology of Δ*mtrA* was visualized by scanning electron microscopy (SEM) (**Figure 1B**), using strains grown for 5 days on solid MS medium. Both the wild-type M145 and the complemented strain 3012/13C-Δ*mtrA* were abundantly covered with loosely coiled spore chains, whereas Δ*mtrA* was covered only by clumps of submerged mycelia (**Figure 1B**), confirming that Δ*mtrA* is defective in the formation of the aerial mycelium, and indicating an essential role for MtrA in normal morphological development.

### Differential Expression of Developmental Genes in Δ*mtrA*

**FIGURE 2:**
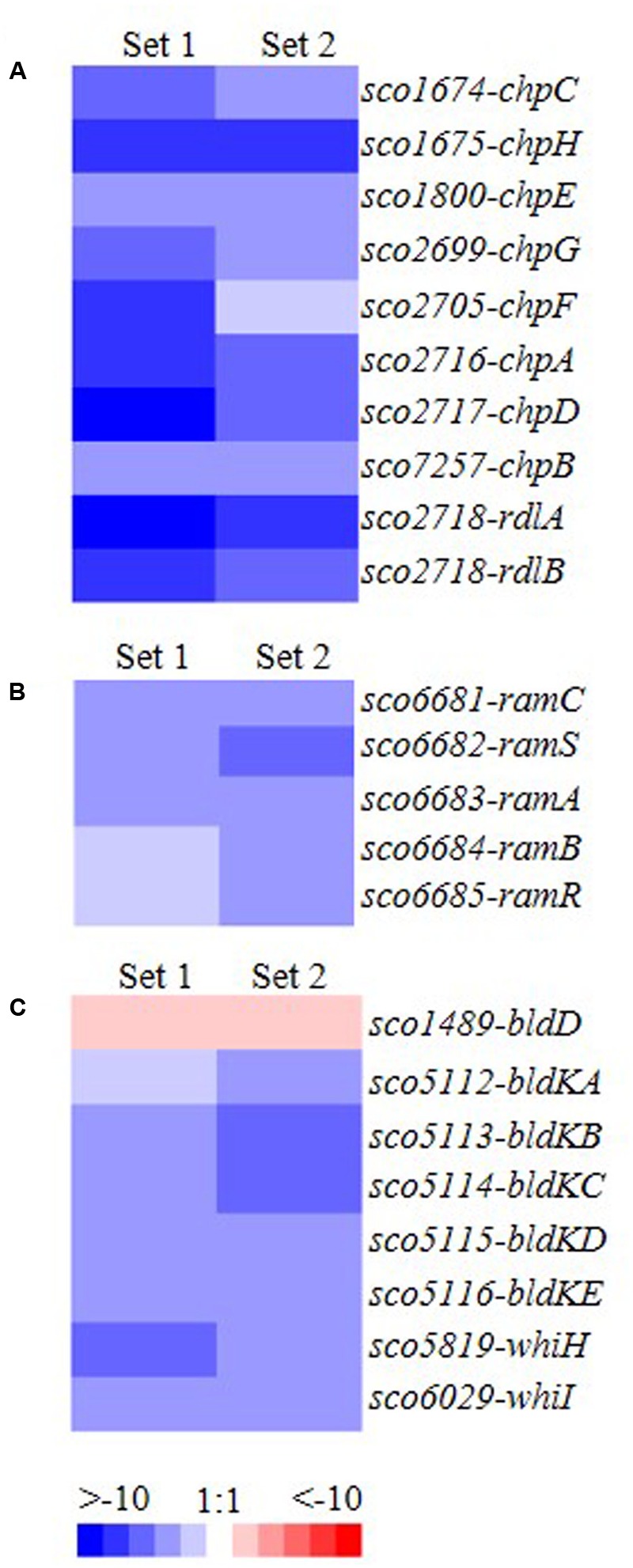
Transcriptomic analysis of genes involved in cellular development and whose expression differs between Δ*mtrA* and wild-type M145. **(A)** *chp* and *rdl* genes (involved in formation of the hydrophobic sheath of the aerial mycelium); **(B)** *ram* genes (involved in production of SapB); **(C)** developmental regulatory genes of the *bld* and *whi* families. Total RNA was extracted from mycelia of M145 and Δ*mtrA* grown for 72 h at 30°C on YBP solid agar, cDNA libraries were prepared, and transcript levels were determined by RNA-Seq. The value for a specific gene was calculated as the log_2_ ratio of the expression level in RPKM (reads per kilobase transcriptome per million mapped reads) of the gene in Δ*mtrA* relative to that in M145, with red indicating an increase in transcript abundance in Δ*mtrA* and blue indicating a decrease.

To investigate the mechanisms underlying the bald phenotype of Δ*mtrA*, we conducted RNA-Seq analysis on wild-type M145 and Δ*mtrA*, using cultures grown for 72 h on solid YBP medium. All eight chaplin genes were significantly down-regulated in Δ*mtrA* compared to M145, as were the two rodlin genes, *rdlA* and *rdlB* (**Figure 2A**). *ramCSAB* and *ramR,* involved in SapB production, were also down-regulated in the mutant (**Figure 2B**). Interestingly, *bldD*, a global transcriptional regulator in *S. coelicolor* (Elliot et al., 1998, 2001; Den Hengst et al., 2010), was overexpressed in Δ*mtrA* (**Figure 2C**), whereas the *bldK* operon, which is responsible for the export of a signal molecule critical for development (Nodwell et al., 1996), was significantly down-regulated, suggesting that MtrA represses *bldD* but activates *bldK*. In addition, *whiH* and *whiI*, the two *whi* genes that play an important role in the late stages of differentiation (Ryding et al., 1998; Ainsa et al., 1999), were down-regulated in Δ*mtrA* (**Figure 2C**), suggesting that MtrA activates *whiH* and *whiI* under the conditions tested. Overall, RNA-Seq analysis indicated that MtrA not only affects genes required for the formation of aerial mycelium but also genes dedicated to spore maturation.

### Analysis of the Expression Patterns of *chp* and *rdl* Genes in Δ*mtrA* during Development

**FIGURE 3:**
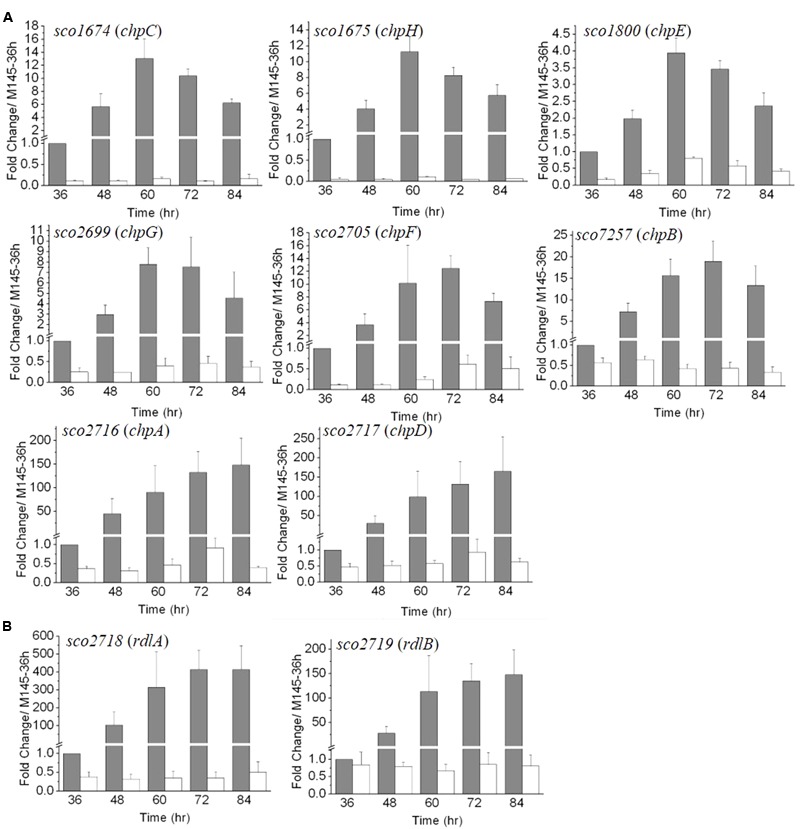
The developmentally regulated expression of chaplin **(A)** and rodlin **(B)** genes in *S. coelicolor* M145 is blocked in Δ*mtrA*. Expression of these morphogen genes was determined by real-time PCR analysis. M145 and Δ*mtrA* were grown on YBP solid medium, and RNA samples were isolated at the indicated times. Expression of *hrdB,* encoding the major sigma factor in M145, was used as an internal control. For each gene, the expression level in M145 at 36 h was arbitrarily set to one. The y-axis shows the fold change in expression levels in M145 (gray bars) and Δ*mtrA* (light bars) over the levels in M145 at 36 h. Results are the means (± SD) of triplet experiments.

The RNA-Seq analysis was conducted at 72 h of growth and was thus a snapshot of the gene expression patterns occurring during the relatively long developmental process. For a more comprehensive view of the role of MtrA during different developmental phases and to validate the RNA-Seq data, real-time PCR was performed. RNA was extracted from M145 and Δ*mtrA* at 12-h intervals over 36–84 h of growth on solid YBP medium, a time period that covers the formation of vegetative mycelium, aerial mycelium, and spores. The expression level of each gene at 36 h in M145 was arbitrarily set to 1. Based on their expression patterns, the eight chaplin genes could be divided into two groups (**Figure 3A**). The first group, comprising *chpB* (*sco7257*), *chpC* (*sco1674*), *chpE* (*sco1800*), *chpF* (*sco2705*), *chpG* (*sco2699*), and *chpH* (*sco1675*), demonstrated peak levels of transcription in M145 between 60 and 72 h, with maximal fold changes in M145 ranging from 3.94 ± 0.44 for *chpE* to 18.96 ± 4.71 for *chpB*, with transcript levels decreasing thereafter. *chpC*, *chpH*, and *chpE* showed the most similar patterns, peaking at 60 h in M145, albeit the induction levels of *chpE* were less than half that of the other two genes (**Figure 3A**). In Δ*mtrA,* expression of genes in this first group was markedly lower, with transcription of *chpC* and *chpH* barely detectable at each of the five time points (**Figure 3A**), indicating that MtrA strongly influences these genes throughout development. The second group of *chp* genes, *chpA* (*SCO2716*) and *chpD* (*SCO2717*), displayed dramatically increasing expression levels throughout the development of M145, with peak expression levels at the last time point (84 h) that were approximately 148.2 and 165.5 times greater than those at 36 h for these genes (**Figure 3A**). However, similar to the genes in group 1, the induction of *chpA* and *chpD* was abolished in Δ*mtrA* (**Figure 3A**).

In M145, *rdlA* and *rdlB* displayed transcription patterns similar to those of *chpA* and *chpD* (**Figure 3B**), with highest transcription levels detected at the last time points (**Figure 3B**). Although *rdlA* and *rdlB* are divergently transcribed, *rdlA* was induced more highly than *rdlB* at each time point, culminating in fold changes of 414.1 ± 105.8 and 147 ± 51.3 at the last time point for *rdlA* and *rdlB*, respectively. However, induction of both genes was blocked in Δ*mtrA*, with expression values of less than 1.0 at all time points (**Figure 3B**), indicating a dramatic effect of MtrA on the developmentally regulated expression of *rdl* genes.

### Transcription of *ram* Genes in Δ*mtrA* during Development

SapB is essential for aerial hyphae formation on rich medium (Willey et al., 1991, 1993), and so, to investigate the impact of MtrA on the expression of genes involved in SapB production, transcription of the *ram* cluster was examined using the same set of RNA samples described above. Whereas the *chp* and *rdl* genes were more highly induced during the late developmental phases when aerial mycelia and spores start to form, *ramC* expression was highest at the first time point (36 h), with expression decreasing to ≤0.2 of the control level for the remaining time points in M145 (Supplementary Figure S1); this finding is in agreement with a previous report, which demonstrated that RamC peaks at 36 h in the wild-type strain when grown on R2YE medium (O’Connor et al., 2002). However, in Δ*mtrA,* transcription of *ramC* was barely detectable at any of the five time points (Supplementary Figure S1). The temporal expression profiles of *ramS*, *ramA*, and *ramB* were similar to those of *ramC* in M145 and Δ*mtrA* (Supplementary Figure S1), with the consistently decreased expression in the mutant providing evidence that these genes form an operon in *S. coelicolor* and that MtrA is essential for their activation, especially at the very early phase of development.

As the *ramC* operon is under the direct control of RamR (Nguyen et al., 2002; O’Connor et al., 2002), we also compared transcription of *ramR* in M145 and Δ*mtrA*. *ramR* expression showed only minor changes in M145, and its expression was only slightly lower in Δ*mtrA*. For example, in Δ*mtrA* at 36 h, *ramR* expression levels were about half the levels found in M145 (Supplementary Figure S1), whereas at the same time point, *ramC* was down-regulated nearly 50-fold in Δ*mtrA* (Supplementary Figure S1). These findings suggest that the dramatically decreased expression of *ramCSAB* is more likely due to the absence of MtrA rather than to the slight reductions in *ramR* expression, and therefore that, compared to RamR, MtrA has a more dominant regulatory effect on *ramCSAB* under the conditions tested.

### Transcription of *bld* Genes in Δ*mtrA* during Development

**FIGURE 4:**
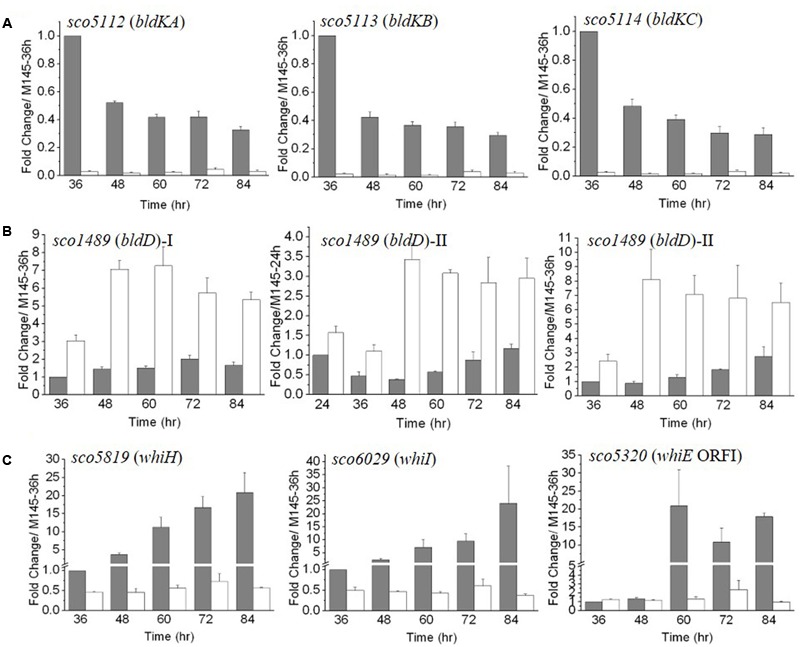
The temporal expression of *bld* **(A,B)** and *whi* **(C)** genes in Δ*mtrA*. Gene expression in M145 and Δ*mtrA* was determined by real-time PCR analysis. RNA samples were isolated from M145 and Δ*mtrA* and analyzed as described for **Figure 3**. The y-axis shows the fold change in expression level in M145 (gray bars) and Δ*mtrA* (light bars) over the level of M145 at 36 or 24 h (arbitrarily set to one). Results are the means (± SD) of triplet experiments.

Our above data indicated that expression of genes involved in the formation of aerial mycelium was blocked in Δ*mtrA*. Notably, these genes are also poorly expressed in most *bld* mutants (Willey et al., 1991; Elliot et al., 2003; Bibb et al., 2012), and our RNA-Seq analysis had indicated that *bldD* and *bldK* genes were differentially expressed in Δ*mtrA* at 72 h growth (**Figure 2**). To determine if the expression of additional *bld* genes was altered in Δ*mtrA*, we used real-time PCR. For the majority of *bld* genes, transcription was either not altered over the five time points examined (*bldA*, *bldB*, and *bldM*) or only slightly reduced at certain time points (*bldC*, *bldG*, *bldH*, and *bldN*) by deletion of *mtrA* (Supplementary Figure S2), suggesting a generally minor effect of *mtrA* on their expression. In contrast, *bldD* and genes of the *bldK* operon exhibited markedly different expression between M145 and Δ*mtrA* throughout development (**Figures 4A,B**). The *bldK* operon encodes an oligopeptide permease, which transports a signal molecule that begins the signaling regulatory cascade required for aerial mycelium formation (Nodwell et al., 1996). *bldKA*, the first gene of the operon, showed a gradually decreasing expression pattern (**Figure 4A**), very similar to that of *ramC* in M145 (Supplementary Figure S1), from the peak level at 36 h to the lowest level of 0.33 ± 0.031 at 84 h, in agreement with the role of BldK during the early developmental stage. However, consistent with the RNA-Seq analysis, no induction of *bldKA* was detected in Δ*mtrA* at any time point, resulting in a 34.5-fold (1 versus 0.029 ± 0.007) difference at 36 h between M145 and the Δ*mtrA* mutant (**Figure 4A**). The expression patterns of *bldKB* and *bldKC* in the two strains were similar to that of *bldKA* (**Figure 4A**). Our data indicate that MtrA activates *bldK,* especially at the early phase of development.

In M145, transcription of *bldD* was fairly constant, ranging from 1.0 to 2.02 ± 0.21 times the level at 36 h (**Figure 4B**); however, its expression was notably higher in Δ*mtrA* at each of the five time points, from 36 to 84 h, with a maximal fold change of 7.26 ± 1.08 for Δ*mtrA* at 60 h versus only 1.50 ± 0.12 for M145 at the same time, suggesting that MtrA represses *bldD* at these time points. Expression of *bldD* normally peaks at the early growth stage (Elliot et al., 1998, 2001; Den Hengst et al., 2010); thus, a second set of RNA samples that included an earlier time point (24 h) was prepared, and expression of *bldD* in the two strains was evaluated relative to the *bldD* expression level at 24 h in M145 (**Figure 4B**, middle panel). In M145, expression of *bldD* at 24 h was approximately twofold higher than at 36 h, consistent with previous reports (Elliot et al., 1998; Den Hengst et al., 2010). In Δ*mtrA,* expression of *bldD* was consistently higher than in M145 at each of the six time points tested and maintained similar, high levels from 48 to 84 h (**Figure 4B**). When the *bldD* expression level in M145 at 36 h was used as the control for the second set of RNA samples, a similar expression pattern was observed as for the first set of RNA samples (**Figure 4B**, compare left and right panels for *bldD*), confirming that MtrA represses *bldD*, especially at the late developmental phase, and revealing MtrA as the first identified regulator of *bldD* other than BldD itself.

### Transcription of *whi* Genes in Δ*mtrA* during Development

Our preliminary transcriptomic data indicated that two *whi* genes, *whiH* and *whiI*, were downregulated in the late stages of development in Δ*mtrA* (**Figure 2C**). For a more comprehensive view of the role of MtrA in *whi* expression, we examined the transcription of the eight canonical *whi* genes throughout development. Transcription of *whiA*, *whiB*, *whiG*, and *whiJ* was not altered, and *whiD* was slightly upregulated in Δ*mtrA* at the five time points tested (Supplementary Figure S3). In contrast, *whiH* and *whiI* showed developmentally associated induction in M145, with peak values at the last time point of 84 h (**Figure 4C**), consistent with a previous report (Ryding et al., 1998; Ainsa et al., 1999). In *S. coelicolor*, *whiH* and *whiI* are critical for cell differentiation (Ryding et al., 1998; Ainsa et al., 1999), and notably, their induction was absent in Δ*mtrA* (**Figure 4C**), suggesting that MtrA has a positive role in the developmentally regulated expression of *whiH* and *whiI*.

The *whiE* gene cluster, responsible for the synthesis of the gray pigment of mature spores, contains eight ORFs, of which *whiE-*ORFI through *whiE-*ORFVII appear to compose an operon that is divergently transcribed from *whiE*-ORFVIII (Davis and Chater, 1990; Kelemen et al., 1998). We chose *whiE*ORF1 and ORFVIII as representatives of the cluster, and in M145, found that these ORFs showed a similar, marked increase in expression at 60 h, with levels remaining well-above control levels through the later time points (**Figure 4C** and Supplementary Figure S3). This induction was not detected in Δ*mtrA*, suggesting that MtrA is required for *whiE* expression, consistent with our observation that the spores of Δ*mtrA* lacked the gray color typical of M145 spores.

In summary, based on our extensive real-time PCR analyses, MtrA not only affects expression of the structural components of the aerial mycelium but also two classes of regulatory genes known to control developmental processes in *S. coelicolor*.

### Binding of MtrA to the Upstream Regions of Developmental Regulatory Genes

**FIGURE 5:**
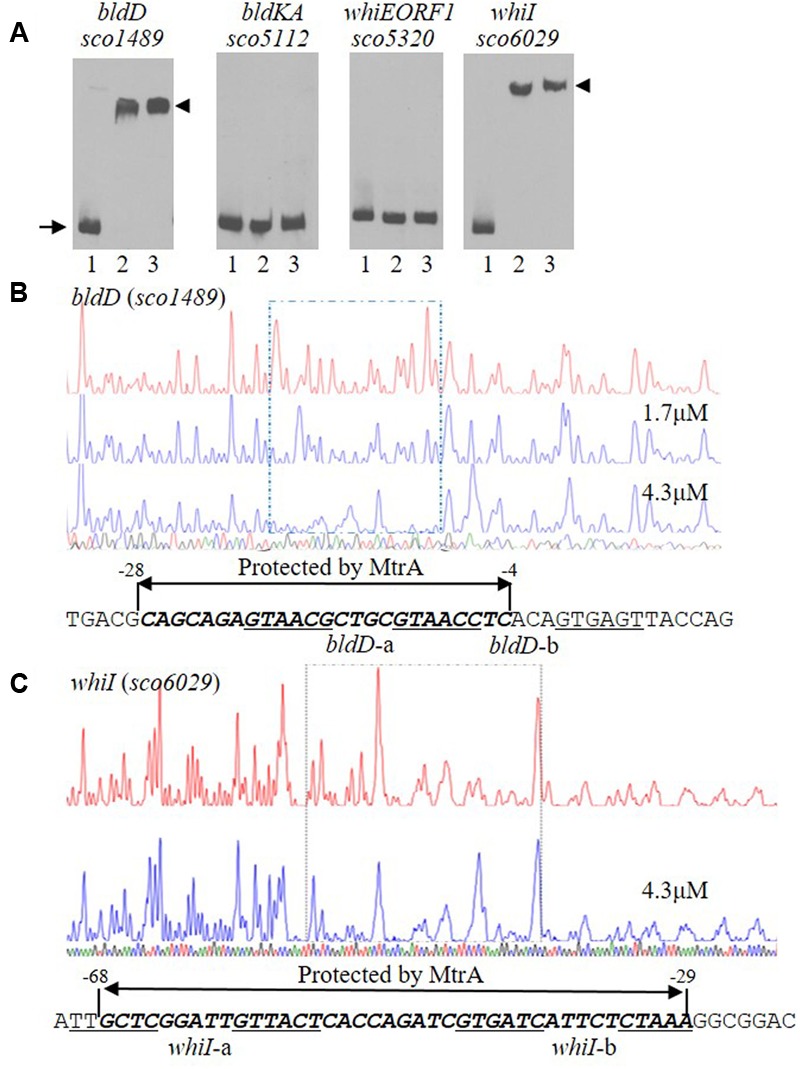
Interactions of MtrA with the upstream regions of *bldD* and *whiI.* **(A)** For EMSAs, a fixed amount of labeled probe containing the intergenic region upstream of the indicated gene was incubated in reactions containing no MtrA (lane 1), or 1.6 or 3.2 μg MtrA (lanes 2 and 3, respectively). The DNA fragments used as probes were amplified by PCR. The length of the probe for each upstream region is: *bldD* (282 bp), *bldKA* (265 bp), *whiE*-ORF1 (267 bp), and *whiI* (293 bp). The arrow indicates the position of free probes, and arrowheads indicate the position of the shifted probes. DNase I footprinting assays of the coding strand of *bldD* **(B)** and *whiI* **(C)**. The upper electropherograms in red are the control reactions without addition of MtrA protein. The reactions including MtrA are shown below in blue. The MtrA-protected regions are indicated by the dotted rectangles. The superimposed electropherograms shows the sequencing reactions. The nucleotide sequence of the MtrA-protected region for each gene is marked by bold italics and a double arrow, and the numbers indicate position relative to the TSP of each gene. The potential MtrA sequence is underlined.

Our above analysis indicated that MtrA controls multiple developmental regulatory genes. To investigate whether MtrA directly regulates these genes, the sequences upstream of *bldD*, *bldKA*, *whiE*-ORF1, and *whiI* were amplified using specific primer pairs (Supplementary Table S2). The resulting DNA fragments, ranging from 250 to 300 bp, were purified, labeled with biotin-11-UTP, and used as EMSA probes. Purified MtrA shifted DNA probes containing the sequences upstream of *bldD* and *whiI* but not those upstream of *bldKA* or *whiE-ORF1* (**Figure 5A**). These *in vitro* analyses suggested that MtrA interacts directly with the promoters of two critical developmental regulatory genes.

### Detection and Validation of the MtrA Binding Sequence

**FIGURE 6:**
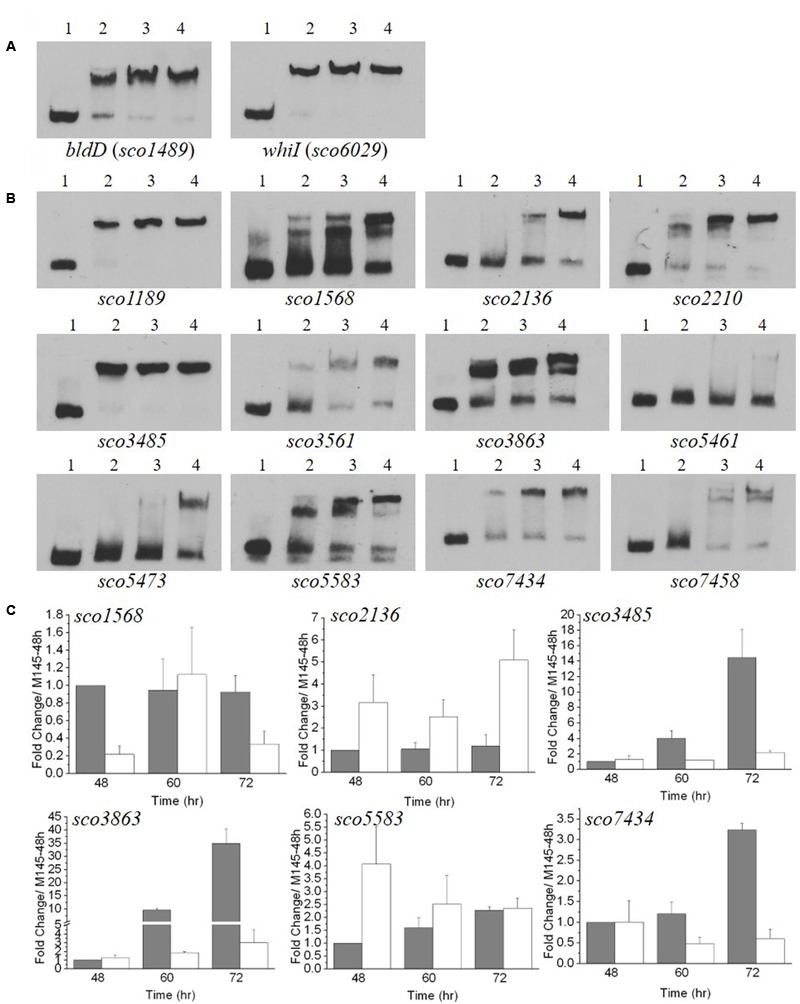
Electrophoretic mobility shift assays (EMSA) and transcriptional analysis of genes with upstream sequences similar to the MtrA_MTB_ recognition sequence. **(A,B)** EMSA analyses with MtrA_SCO_ and 59-bp oligonucleotides containing sequences similar to the MtrA_MTB_ recognition sequence. A fixed amount of labeled oligonucleotide was incubated in reactions containing no MtrA (lane 1), or 1.6 or 3.2, or 4.8 μg MtrA (lanes 2, 3, and 4, respectively). **(A)** Analysis with sequences upstream of *bldD* and *whiI* that were protected by MtrA_SCO_ in DNase I footprinting (see **Figure 5**). **(B)** Analysis with sequences identified in a genome-wide search for matches to the MtrA_MTB_ consensus recognition sequence. The sequences were located upstream of the named genes. **(C)** Transcriptional analysis of potential MtrA_SCO_ target genes in the Δ*mtrA* mutant. M145 and Δ*mtrA* were grown on YBP solid medium, and RNA samples were isolated at the indicated times. Expression of *hrdB,* encoding the major sigma factor in M145, was used as an internal control. The y-axis shows the fold change in expression level in M145 (gray bars) over the level of Δ*mtrA* (light bars) at each time point, which was arbitrarily set to one. Results are the means (± SD) of triplet experiments.

To map the precise sequences protected by MtrA, we performed DNase I footprinting assays using FAM-labeled probes. For *bldD*, the region from -276 to +6 with respect to its TSP (Elliot et al., 1998; Elliot and Leskiw, 1999) was used as template, and MtrA protected the sequence from -28 to -4 in the sense strand (**Figure 5B**), which overlaps the -10 region (Elliot et al., 1998; Elliot and Leskiw, 1999). For *whiI*, we used as template a 293-bp fragment from positions -213 to +80 relative to its TSP (Ainsa et al., 1999), and MtrA protected a 40-nucleotide region, from positions -68 to -29, that overlaps the -35 sequence of the *whiI* promoter (**Figure 5C**). To test the ability of MtrA to bind the protected sequence of *bldD* in EMSAs, a short oligonucleotide containing this region was used as a probe and was shifted completely by MtrA even with the lowest amount of protein tested (**Figure 6A**). Similarly, MtrA also retarded completely a short probe containing the protected sequence upstream of *whiI* (**Figure 6A**).

MtrA_SCO_ has 75% amino acid identity to MtrA_MTB_ and 69% amino acid identity to MtrA_CGL_ (Hoskisson and Hutchings, 2006). The consensus sequences recognized by MtrA_MTB_ and MtrA_CGL_ have been characterized *in vitro* (Brocker et al., 2011) and *in vivo* (Galagan et al., 2013), respectively, revealing a similar, loosely conserved sequence composed of two 6-bp direct repeats (Supplementary Figure S4). These repeats are biased toward A/T for MtrA_CGL_ and toward G/C for MtrA_MTB_ (Brocker et al., 2011; Galagan et al., 2013), possibly owing to the lower G+C content of the genome of *C*. *glutamicum* compared to that of *M*. *tuberculosis* (Cole et al., 1998; Kalinowski et al., 2003). Because of the higher sequence identity of MtrA_SCO_ to MtrA_MTB_ and the similar G+C content of their genomes, we hypothesized that the consensus sequence recognized by MtrA_SCO_ would more closely resemble the one recognized by MtrA_MTB_. An examination of the sequences protected by MtrA_SCO_ upstream of *bldD* (**Figure 5B**) and *whiI* (**Figure 5C**) revealed matches to the MtrA_MTB_ consensus, suggesting the two proteins have similar binding motifs.

The genome of *S. coelicolor* M145 was searched using the MtrA_MTB_ binding consensus sequence (Galagan et al., 2013), and multiple potential recognition sites for MtrA_SCO_ were discovered (data not shown). The sites with high scores were selected, and the ability of MtrA_SCO_ to bind these sites was tested using short, oligonucleotide probes. In EMSA analysis, almost complete shifting was observed with probes for regions upstream of *sco1189* and *sco3485,* with the least amount of MtrA protein tested (**Figure 6B**). With increasing amounts of MtrA, increased shifting was detected with probes for regions upstream of *sco1568*, *sco2136*, *sco2210*, *sco3561*, *sco3863*, *sco5583*, *sco7434*, and *sco7458*. However, probes for regions upstream of *sco5461* and *sco5473* were shifted only with the highest amounts of MtrA (**Figure 6B**). To test whether MtrA regulates these genes, transcription of the six genes was evaluated at 48, 60, and 72 h, with the transcription of the gene in M145 at 48 h arbitrarily set to one (**Figure 6C**). Expression of four genes, *sco1568*, *sco3485*, *sco3863*, and *sco7434*, was significantly down-regulated in Δ*mtrA* in at least one time point (**Figure 6C**), whereas *sco2136* and *sco5583* were up-regulated in Δ*mtrA* in at least one time point (**Figure 6C**), confirming that MtrA regulates these genes and consistent with the EMSA data.

### Mutational Analyses of the MtrA_SCO_ Consensus Binding Site

**FIGURE 7:**
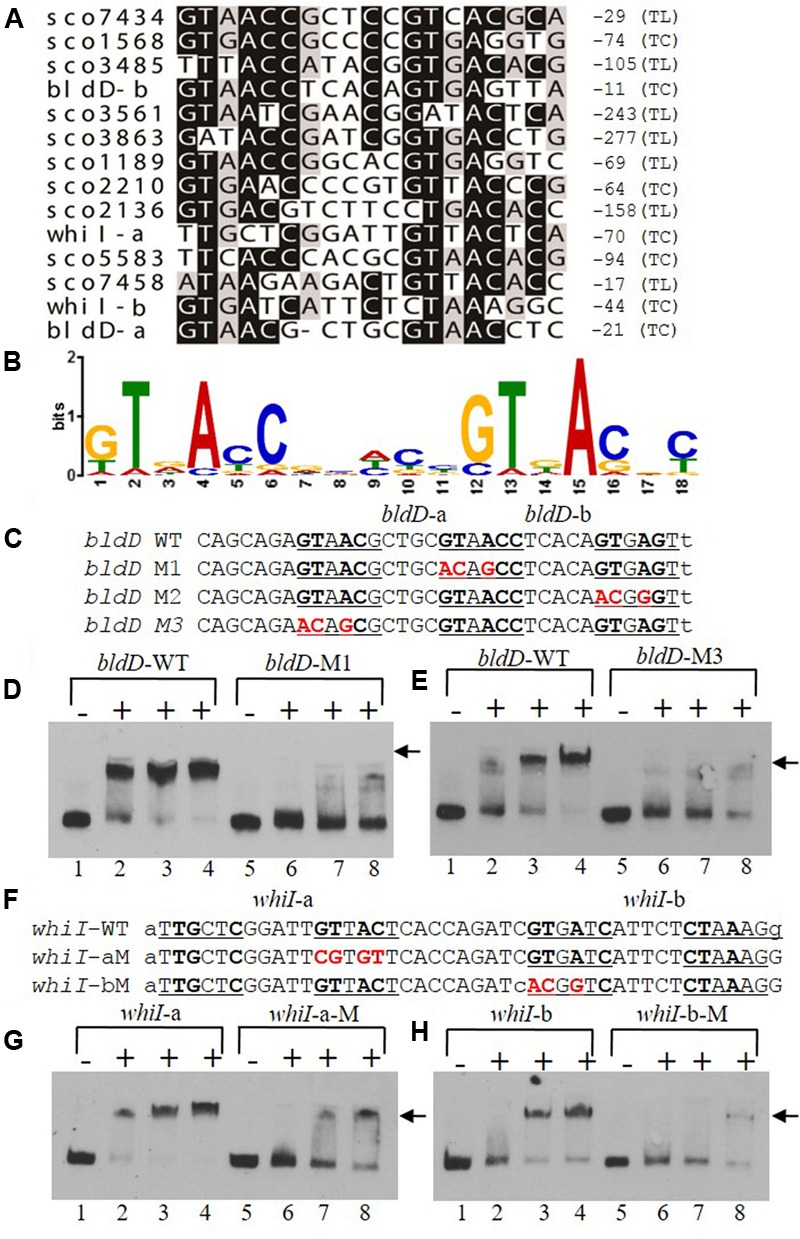
A consensus binding sequence for MtrA_SCO_ and mutational analysis of predicted binding sites in the *bldD* and *whiI* promoters. **(A)** Alignment of MtrA-shifted sequences (see **Figure 6**), with conserved nucleotides indicated by a darkened background. Numbers at the right indicate distance from putative translational start site (TL) or transcriptional start site (TC). Two putative sites are present upstream of *bldD* (*bldD*-a and *bldD*-b) and *whiI* (*whiI*-a and *whiI*-b). **(B)** Consensus sequence for MtrA recognition based on alignment in **(A)** and containing two direct repeats of approximately six nucleotides. **(C–H)** Mutational analyses of putative MtrA binding sites in the promoters of **(C–E)** *bldD* and **(F–H)** *whiI*. Mutagenized bases are shown in red, and probes with these mutations were compared with the wild-type sequence for binding to MtrA in EMSAs. **(D,E,G,H)** Reactions were carried out with the addition of no MtrA (lanes 1, 5); 2.1 μg MtrA (lanes 2, 6); 4.2 μg MtrA (lanes 3, 7); or 6.3 μg MtrA (lanes 4, 8). Arrowheads indicate the positions of the shifted probes.

To determine a consensus binding sequence for MtrA_SCO_, the 13 probes, including those for *bldD* and *whiI*, that had stronger affinity to MtrA_SCO_ in EMSA analysis (**Figures 6A,B**) were used as input sequences for MEME analysis (**Figure 7A**). An 18-nucleotide consensus sequence with two, imperfect direct repeats of five nucleotides was revealed for MtrA_SCO_ binding (**Figure 7B**), a sequence very similar to the consensus binding sequences for MtrA_MTB_ and MtrA_CGL_, although with some variation in the degree of conservation for each nucleotide (Supplementary Figure S4). To validate the role of the consensus sequence in MtrA_SCO_ binding, we performed EMSAs using oligonucleotide probes containing point mutations in the repeat motifs upstream of *bldD* (**Figure 7C**). Close analysis of the sequence had revealed two overlapping sets of repeats that shared one half-site, and in contrast to the other sites (**Figure 7A**), the repeats in *bldD*-a are separated by one less nucleotide. Mutation of as few as three nucleotides in the central half-site markedly decreased MtrA binding in comparison to that observed with the native sequence (**Figure 7D**). Mutation of the three conserved nucleotides in the rightmost half-site only slightly reduced MtrA binding (Supplementary Figure S5), whereas mutation of three of the four conserved nucleotides in the left most motif markedly reduced MtrA binding (**Figure 7E**), suggesting that the left set of repeats may be more important for MtrA binding to the *bldD* promoter.

Two potential MtrA binding sites were identified upstream of *whiI* in the MtrA-protected sequence (**Figure 7F**). The first site, designated *whiI*-a, includes positions -69 to -53 and the second site, designated *whiI*-b, includes positions -43 to -27, relative to the TSP of *whiI* (**Figure 7F**). EMSAs suggested that MtrA has stronger affinity for *whiI*-a than for *whiI*-b (**Figures 7G,H**), possibly because *whiI*-a has more conserved nucleotides in the direct repeats than does *whiI*-b (**Figures 7A,F**). Mutation of three or four of the conserved nucleotides within *whiI*-a or *whiI*-b probes severely reduced MtrA binding (**Figures 7G,H**). These data suggest that the *whiI* promoter contains more than one MtrA binding site and that the conserved nucleotides contribute to MtrA binding. Collectively, these data support the consensus sequence identified for MtrA binding.

### Mutation of the *mtrA* Homologs in *S. venezuelae* and *S. lividans* Leads to a Conditional Bald Phenotype

**FIGURE 8:**
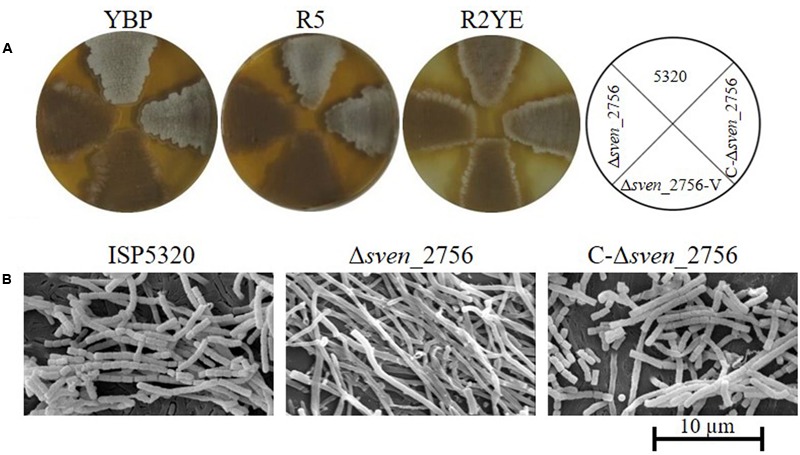
Requirement of the *mtrA* homolog *sven_2756* for the formation of aerial hyphae in *S. venezuelae*. **(A)** Phenotypes of the *S. venezuelae* wild-type strain ISP5320, the deletion mutant Δ*sven_2756*, and the *sven_2756*-complemented strain C-Δ*sve_2756* grown at 30°C on solid YBP (5 days), R5 (5 days), and R2YE media (84 h). **(B)** SEM images of *S. venezuelae* ISP5320, Δ*sven_2756*, and C-Δ*sven_2756* strains after growth on YBP agar for 84 h. Images reveal the classic ‘bald’ phenotype for the deletion mutant and normal aerial hyphae for the wild-type and complemented strains. The scale bar is 10 μm.

BLAST analysis indicated that proteins with high similarity to MtrA_SCO_ are present in other *Streptomyces* species, including *S. griseus*, *S. venezuelae*, *S. clavuligerus*, *S. scabies*, and *S. avermitilis*^1^, reaching 98–100% amino acid identity. To investigate the functional conservation of MtrA in other *Streptomyces* species, the coding sequence of *sve_2756*, the *mtrA* homolog in *S. venezuelae*, was deleted in the wild-type strain ISP5320, generating Δ*sve_2756*. Δ*sve_2756* displayed a bald phenotype, especially on rich medium, with significantly delayed formation of aerial mycelium on R5, R2YE, and YBP media (**Figure 8A**). On YBP solid medium, growth of aerial mycelium was observed as early as 48 h for ISP5320; however, Δ*sve_2756* remained bald even after 8 days of growth under the same conditions (**Figure 8A**). The complemented strain C-Δ*sve_2756* was generated using pCom-2756_SV E_, and as expected, this complementation restored the morphological defect (**Figure 8A**), confirming that the bald phenotype of Δ*sve_2756* is a direct result of *sve_2756* mutation and indicating that SVE_2756 is required for normal development in *S. venezuelae*. However, some mutant strains of *S. venezuelae* appear bald, but in fact produce precocious hypersporulation rather than fail to form aerial hyphae (Tschowri et al., 2014). To determine whether the bald phenotype of Δ*sve_2756* was due to defective formation of aerial mycelium, its morphology was observed by SEM (**Figure 8B**), using strains grown for 84 h on YBP solid medium. Both the wild-type ISP5320 and the complemented strain C-Δ*sve_2756* were abundantly covered with straight spore chains, whereas Δ*sve_2756* was covered only by vegetative mycelia (**Figure 8B**), confirming that Δ*sve_2756* is defective in the formation of the aerial mycelium, and indicating an essential role for SVE_2756 in normal morphological development in *S. venezuelae*.

*Streptomyces lividans* is nearly identical to *S. coelicolor* in sequence, but has obvious differences in morphology and physiology (Lewis et al., 2010). To investigate the role of the *mtrA* homolog *sli_3357* in *S. lividans*, the deletion mutant Δ*sli_3357* was generated in strain 1326. Strain Δ*sli_3357* exhibited slightly delayed formation of aerial mycelium on MS medium (Supplementary Figure S6A), with a more obvious delay on R2YE and YBP media (Supplementary Figure S6A). In the complemented strain C-Δ*sli_3357,* generated using pCom-3013_SCO_, growth of aerial mycelium was restored to levels comparable to that of the parental strain 1326 (Supplementary Figure S6A). SEM of cultures grown on YBP solid medium revealed that the wild-type strain 1326 and complemented strain C-Δ*sli_3357* were covered with abundant, straight or slightly coiled spore chains, whereas only fragments of submerged mycelium were observed for Δ*sli_3357* (Supplementary Figure S6B), confirming that Δ*sli_3357* is defective in the growth of aerial mycelia and indicating SLI_3357 is required for development in *S. lividans*. Altogether, these results suggest that MtrA homologs have a similar and essential role in the normal development of *Streptomyces* species.

## Discussion

### MtrA Is a New Member of Developmental Regulators in *Streptomcyes*

Our study showed that mutation of the response regulator gene *mtrA* leads to the bald phenotype characteristic of *bld* mutants, suggesting that MtrA is a new developmental regulator in *S. coelicolor*. We further determined that MtrA controls multiple genes critical for development, most notably *bldD*, *bldK*, *whiH*, and *whiI*; additionally, *bldD* and *whiI* were characterized as MtrA targets through *in vitro* analysis. As transcription of *chp*, *rdl*, and *ram* genes was also markedly influenced by deletion of *mtrA* in our study, we investigated whether MtrA regulates these genes directly or indirectly. Our preliminary data showed that MtrA_SCO_ interacts with the upstream sequence of several *chp*, *rdl*, and *ram* genes (Supplementary Figure S7), implying potentially direct control of MprA_SCO_ over these structural components of aerial mycelium. Although the data in this study were obtained mostly from *in vitro* analysis, such as EMSAs, our data are consistent with a recent study carried out in *S. venezuelae* that characterized the *in vivo* targets of MprA_SV E_ using the Chip-Seq assay (Som et al., 2017). Som et al. (2017) reported that *whiI*, which we identified as a direct target of MtrA_SCO_, was one of the four *in vivo* MtrA targets with a statistically significant high enrichment value in *S. venezuelae* using Chip-Seq. Moreover, several *bld* genes (*bldG*, *bldH*, *bldM*, and *bldN*), *whi* genes (*whiB*, *whiD*, *whiG*, and *whiH*), *chp* genes (*chpH*, *chpE*, *chpG*, *chpF*, *chpG*, and *chpD*), *rdlAB*, and *sapB* displayed moderate to low levels of enrichment in Chip-Seq analysis, indicating that these genes are potential MtrA targets in *S. venezuelae*, consistent with many of our *in vitro* findings for *S. coelicolor*. However, *bldD*, which we found to be a direct target of MtrA through *in vitro* assays, was not identified as an MtrA target in *S. venezuelae*, possibly due to species differences.

**FIGURE 9:**
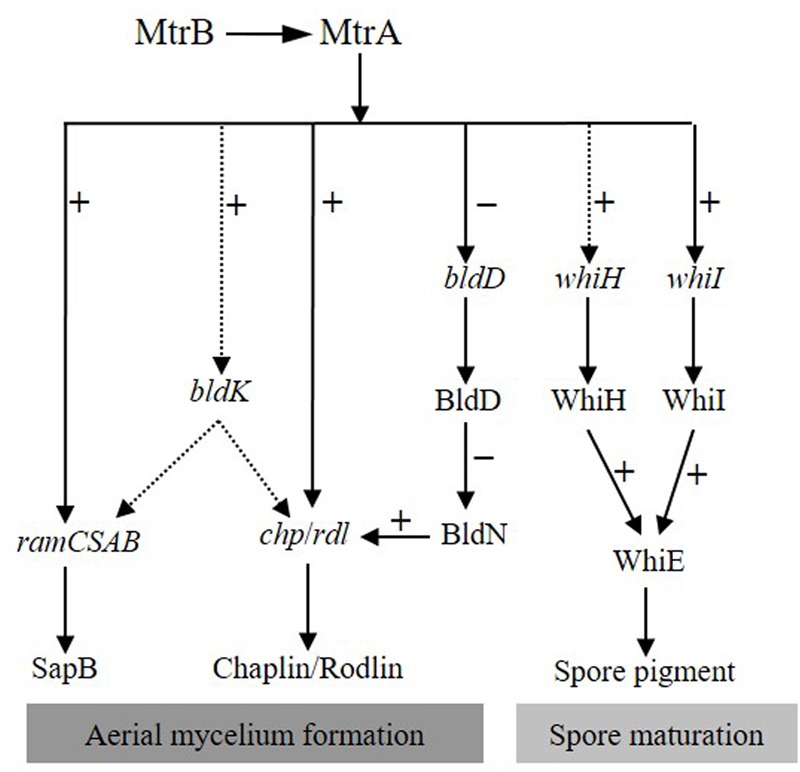
Proposed model of the MtrA regulatory network governing development and differentiation in *S. coelicolor*. In response to a currently unidentified environmental trigger, MtrA is activated by MtrB phosphorylation, and then the activated MtrA activates *chp*, *rdl*, and *ram* genes and the *bldK* cluster, the latter of which may also have a positive regulatory effect on the expression of *ram*, *rdl*, and *chp* genes. In contrast, MtrA represses *bldD*, allowing activation of *bldN*, a sigma factor gene repressed by BldD-c-di-GMP, and BldN then activates *chp* and *rdl* genes, further amplifying the effect of MtrA on the regulation of aerial mycelium formation. MtrA regulates the expression of *whiI* and *whiH*, both of which are required for expression of *whiE*, thus impacting differentiation through the *whiIHE* pathway. Solid and dotted arrows indicate direct and indirect regulation, respectively. Minus (–) and plus (+) signs indicate a negative or positive role in the regulation of the downstream gene.

Based on the analyses for MtrA in *S. coelicolor* and *S. venezuelae*, we propose a simple model for the MtrA regulatory network in governing development and differentiation in *S. coelicolor* (**Figure 9**). In this model, on receiving unknown environmental signals, the sensor kinase MtrB phosphorylates its cognate regulator MtrA, and the phosphorylated MtrA activates the *chp* and *rdl* genes that make up the components of aerial mycelium, and the *ramCSAB* operon at the early phase, resulting in the production of SapB and promoting the formation of aerial mycelium. MtrA also initiates, although possibly indirectly, the transcription of the *bldK* cluster, thus enabling transport of an early signal molecule into the cell (Nodwell et al., 1996), which also has a positive effect on SapB production and expression of *chp* and *rdl* genes through as yet unknown mechanisms. MtrA directly represses *bldD*, and the resulting lower levels of BldD allow activation of BldN whose targets include *chp* and *rdl* genes (Elliot et al., 2003; Bibb et al., 2012). The bald phenotype of Δ*mtrA* is thus likely a combined effect of the deeply blocked expression of chaplins and rodlins, which are the structural components of aerial mycelium; the minimal production level of SapB, a protein that assists in the erection of vegetative mycelium into the air; the blocked transcription of *bldK,* which results in the failed import of the early signal molecule critical for cellular development; and the overexpression of *bldD,* which may help maintain the mycelium in the vegetative phase. Additionally, MtrA also regulates *whiI* directly and *whiH* indirectly, and as expression of both genes is essential for activation of *whiE* (Kelemen et al., 1998), MtrA thus impacts *Streptomyces* differentiation through the *whiIHE* pathway (**Figure 9**).

### MtrA Is a Transcriptional Regulator of *bldD*

*Streptomyces* development is a complex process controlled by *bld* and *whi* genes, which form a developmentally regulatory network. BldD seems to be a master regulator of this network, with the BldD-c-di-GMP complex directly controlling *bldA*, *bldC*, *bldH*, *bldM*, *bldN*, *whiB*, and *whiG* (Den Hengst et al., 2010), and indirectly controlling *whiH*, *whiI*, and *whiE* (Bush et al., 2015, 2016). BldD is autoregulatory, repressing its own expression (Elliot et al., 1998), but no other regulators of *bldD* were previously identified. We found that MtrA can bind the region upstream of *bldD*, that mutation of an MtrA consensus target sequence reduces MtrA binding to this region, and that deletion of *mtrA* results in enhanced expression of *bldD*, revealing MtrA as a second regulator of *bldD*. Previous studies have shown that, when wild-type *S. coelicolor* is grown on solid R5 or R2YE, two types of media with essentially the same components, *bldD* expression peaks at the very early growth phase (about 14 to 15 h), decreases by 24 h, although still remaining relatively high, and then becomes very low by 48 h (Elliot et al., 1998; Den Hengst et al., 2010). Consistent with these reports, we observed decreased *bldD* expression between 24 and 48 h in M145. However, the expression pattern of *bldD* after 48 h has not been previously reported, and unexpectedly, we detected increased expression at later time points compared to the level at 48 h. Of particular interest, the *bldD* expression level in Δ*mtrA* was consistently higher than in M145 at all the time points tested, with the highest expression levels detected in the mutant from 48 h to 84 h, suggesting that *bldD* is derepressed, especially at the late growth phase in Δ*mtrA*. As a global regulator, BldD represses genes essential for morphological development and sporulation (Elliot et al., 2001; Den Hengst et al., 2010; Tschowri et al., 2014), and it was proposed that BldD represses premature expression of *bld* target genes during vegetative growth (Elliot et al., 2001; Tschowri et al., 2014). Notably, although both BldD (Elliot et al., 1998) and MtrA repress *bldD* transcription, *bldD* was significantly derepressed at the early stage in a *bldD* mutant (Elliot et al., 1998), whereas derepression of *bldD* was most evident at the late phase in Δ*mtrA*, potentially reflecting different regulatory roles. Interestingly, the MtrA-protected sequence overlaps the BldD sites in the *bldD* promoter (Elliot et al., 2001; Den Hengst et al., 2010), suggesting that BldD and MtrA may interact to control this gene in *S. coelicolor*.

### MtrA_SCO_ Has an Important but a Different Role, Compared with MtrA_MTB_ and MtrA_CGL_

MtrA has been primarily studied in *M. tuberculosis* and *C*. *glutamicum*, displaying differing but critical roles in these organisms (Zahrt and Deretic, 2000, 2001; Moker et al., 2004). In addition to its essential role in cell viability, MtrA_MTB_ is also implicated in modulating cell proliferation (Fol et al., 2006), by sequestering *oriC* (Purushotham et al., 2015). The primary targets of MtrA_MTB_ include *dnaA*; *fbpB,* which encodes the major secreted immunodominant antigen Ag85B; and *rpfB,* encoding the resuscitation-promoting factor (Li et al., 2010; Rajagopalan et al., 2010; Sharma et al., 2015). The contribution of MtrA_CGL_ is manifested in cell morphology, resistance to antibiotics, and osmoprotection (Moker et al., 2004), and its target genes have diverse functions, including genes encoding a putative cell wall peptidase, transporters, and membrane proteins (Moker et al., 2004; Brocker and Bott, 2006; Brocker et al., 2011). Our study revealed a unique function for MtrA in *Streptomyces*, where it has a major role in the developmental life cycle, controlling multiple genes involved in the formation of the aerial mycelium.

In a system-wide study to characterize the global regulatory network of transcription factors (TFs) in *M. tuberculosis*, target sequences for TFs were identified by Chip-seq analysis, and for MtrA_MTB_, a conserved recognition sequence composed of two imperfect direct repeats of six nucleotides was deduced from more than sixty target sequences (Galagan et al., 2013). A binding motif similar to that of MtrA_MTB_ but more biased to A/T was deduced through *in vitro* analysis for MtrA_CGL_ (Brocker et al., 2011). We identified similar direct repeats of 5–6 nucleotides, G/T-T-G/A-A-C-C-NNNNN-G-T-G/T-A-C-N, separated by five nucleotides, in regions bound by MtrA_SCO_, including a site that overlaps the -10 region in the *bldD* promoter, potentially explaining how MtrA represses *bldD*. For *whiI*, two potential MtrA_SCO_ sites, *whiI*-a and *whiI*-b, were found upstream of *whiI*, with *whiI*-b overlapping the -35 region and *whiI-a* upstream of the -35 region (from positions -68 to -53). Given that binding to the *whiI*-b site could potentially inhibit transcription, the positive regulatory effect of MtrA over *whiI* expression implies that *whiI-a* maybe the major binding site for MtrA, and notably, EMSAs analysis indicated that MtrA appears to have a higher affinity for *whiI-a* than for *whiI-b*. In addition, as noted above, our conclusion that *whiI* is a direct target of MtrA is further supported by the recent *in vivo* study on MtrA targets in *S. venezuelae* (Som et al., 2017). Also consistent with the *S. venezuelae* study, we detected interactions between MtrA_SCO_ and upstream regions of *chp* genes, *rdl*, and *ramC*, and found loosely conserved MtrA sites upstream of these genes (Supplementary Figure S7). Additionally, our RNA-Seq and real-time PCR analyses on the parental strain and Δ*mtrA* indicate that these genes are positively regulated by MtrA. Although transcription of *whiB* and *whiG* did not appear to be altered in Δ*mtrA* under the conditions used in our study (Supplementary Figure S3), potential MtrA sites were predicted upstream of these two genes (Supplementary Table S3), consistent with the *in vivo* study in *S. venezuelae*, which identified *whiB* and *whiG* as MtrA targets (Som et al., 2017).

### Any Other Role(s) of MtrA in *Streptomyces*?

In addition to its role in development, are there any other functions for MtrA in *Streptomyces*? Our preliminary analyses indicate that production of the signature blue pigment actinorhodin in Δ*mtrA* differs from that in *S. coelicolor* M145 (**Figure 1A**), and we therefore hypothesize that, in addition to a key role in regulating development, MtrA also contributes to the regulation of secondary metabolism in *S. coelicolor*. Furthermore, a genome-wide search of *S. coelicolor* revealed 100s of intergenic sequences highly similar to the MtrA_SCO_ consensus binding sequence (Supplementary Table S3), suggesting that MtrA is a global regulator in *S. coelicolor,* although the function of many of these genes is not yet known. Additional studies on the intriguing roles of this TCS are ongoing.

## Author Contributions

XP conceived and supervised the studies, and wrote the paper; PZ, LW, and YZ performed all experiments; ML carried out bioinformatics analysis; GC, YW, X-LC, MT, and XP analyzed the data.

## Conflict of Interest Statement

The authors declare that the research was conducted in the absence of any commercial or financial relationships that could be construed as a potential conflict of interest.

## Footnotes

1 http://www.streptomyces.org.uk/
