# Supplementary material for: Deletion of MtrA Inhibits Cellular Development of Streptomyces coelicolor and Alters Expression of Developmental Regulatory Genes
Source: Front Microbiol. 2017 Oct 16;8:2013. doi: 10.3389/fmicb.2017.02013 (PMC5650626; doi:10.3389/fmicb.2017.02013)
Supplement: Supplementary file 1 [file Table_1.PDF]

Table S1. Bacterial strains and plasmids used in this study

Strain or plasmid	Description	Reference
**Strains**		
S. coelicolor		
M145	Wild-type, SCP1$^-$ SCP2$^-$	(Kieser, 2000)
Δ*mtrA*	*mtrA::aac(3)IV*	This study
3012/13C-Δ*mtrA*	Δ*mtrA* complemented with pCom-3012/13$_{SCO}$	
3013C-Δ*mtrA*	Δ*mtrA* complemented with pCom-3013$_{SCO}$	This study
S. lividans		
1326	Wild-type	(Kieser, 2000)
Δ*sli_3357*	*sli_3357::aac(3)IV*	This study
C-Δ*sli_3357*	Δ*sli_3357* complemented with pCom-3013$_{SCO}$	This study
S. venezuelae		
ISP5230	Wild-type	(Glazebrook et al., 1990)
Δ*sven_2756*	*sven_2756::aph*	This study
C-Δ*sven_2756*	Δ*sven_2756* complemented with pCom-2756$_{SVEN}$	This study
E. coli		
XL1-Blue MR	Strain used for genomic library construction	Stratagene
Novablue	General cloning strain	Novagen
Transetta BL21	Strain used for protein expression	Sangon
ET12567(pUZ8002)	Strain used for conjugation between *E. coli* and *Streptomyces*	(Kieser, 2000)
BW25113	Strain used for PCR-targeting	(Gust et al., 2003)
**Plasmids**		
SuperCos I	Vector for genomic library costruction	Stratagene
pMD18-T	General cloning vector	Takara
pCR-BLUNT	General cloning vector	Invitrogen
pJTU1278	*E. coli-Streptomyces* shuttle vector	(He et al., 2010)
pMu-2756	pJTU1278 carrying a 5.3 kb fragment with a 2.1 kb left flanking sequence, a 1.2 kb kanamycin cassette, and a 2.0 kb right flanking sequence, of the deleted sequence of *sven_2756*	This study
pEX-*mtrA*	*mtrA* expression plasmid	This study
pMS82	*Streptomyces* integrative vector with hygromycin resistance	(Gregory et al., 2003)
pCom-3013$_{SCO}$	pMS82 with the 687-bp coding sequence of *mtrA* and 792-bp upstream sequence from *S. coelicolor* M145	This study
pCom-3012/13$_{SCO}$	pMS82 with the coding sequence of *mtrA*, *mtrB*, and upstream sequence from *S. coelicolor* M145	
pCom-2756$_{SVEN}$	pMS82 with the 678-bp coding sequence of *sven_2756* and 822-bp upstream sequence from *S. venezuelae* ISP5230	This study

REFERENCES

Glazebrook, M.A., Doull, J.L., Stuttard, C., and Vining, L.C. (1990). Sporulation of Streptomyces venezuelae in submerged cultures. *J Gen Microbiol* 136**,** 581-588.

Gregory, M.A., Till, R., and Smith, M.C. (2003). Integration site for Streptomyces phage phiBT1 and development of site-specific integrating vectors. *J Bacteriol* 185**,** 5320-5323.

Gust, B., Challis, G.L., Fowler, K., Kieser, T., and Chater, K.F. (2003). PCR-targeted Streptomyces gene replacement identifies a protein domain needed for biosynthesis of the sesquiterpene soil odor geosmin. *Proc Natl Acad Sci U S A* 100**,** 1541-1546.

He, Y., Wang, Z., Bai, L., Liang, J., Zhou, X., and Deng, Z. (2010). Two pHZ1358-derivative vectors for efficient gene knockout in streptomyces. *J Microbiol Biotechnol* 20**,** 678-682.

Kieser, T., Bibb, M. J., Buttner, M.J., Chater, K. F., and Hopwood, D.A (ed.). (2000). *Practical Streptomyces Genetics.* Norwich: John Innes Foundation
