# Supplementary material for: Deletion of MtrA Inhibits Cellular Development of Streptomyces coelicolor and Alters Expression of Developmental Regulatory Genes
Source: Front Microbiol. 2017 Oct 16;8:2013. doi: 10.3389/fmicb.2017.02013 (PMC5650626; doi:10.3389/fmicb.2017.02013)
Supplement: Supplementary file 2 [file Table_2.PDF]

Table S2. Primers used in this study

Primers	Sequence (5′-3′)
**For fishing library cosmid**	
sco3013-hybrid-foward	TCTAGAGTGACCTTCATCCGTTCCTG
sco3013-hybrid-reverse	ACTAGTACCCGCCTGGTCAACGT
**For *mtrA* mutation**	
sco3013-Target-F	GGCCTCGCTGTGTGCCAGGTCACGCCAGGTAAC GATTAGCTAATGATTCCGGGGATCCGTCGACC
sco3013-Target-R	GCCCCGGACCCGGGCGCCGAAGCGGCACTGTCC CTGGCCATGTCATGTAGGCTGGAGCTGCTTC
**For *mtrA* mutation verification**	
Δ*mtrA*-Confirm-F	GTTGACCTGCTTGCCGATGA
Δ*mtrA*-Confirm-R	CCTGACGGCATACGAG
**For genetic complementation**	
sco3013-Com-F	CCAAGCTTTCGCCGGGACCGGTGA (HindIII)
sco3013-Com-R	CCAAGCTTCGAAGCGGCACTGTCCCT (HindIII)
sven_2756 Com-F	CCAAGCTTTCGGGGACGAGGTACCGCTGGG （HindIII）
sven_2756 Com-R	CCAAGCTTGGGCTTCGGAGCAGCACTGCCTGT （HindIII）
**For protein expression**	
mtrA-Exp-F	CGCCATATGATGTCGTTTATGAAGGGACGAG (NdeI)
mtrA-Exp-R	CCCAAGCTTTCAGCTCGGTCCGGCCTTGTAGCCG (HindIII)
**For EMSA assay**	
SCO1489 promotor F	GCCTCACGGGACGGACCTTAAAGGAC
SCO1489 promotor R	GGACATATGGCTCCCCGGACAAGGTG
SCO5112 promotor F	GTCGTCCCCGATGGCAGGTGTAG
SCO5112 promotor R	TCGTCATGATTCGTCCATTTCAC
SCO5320 promotor F	CGTCAGGGATGCGTCAGGAGTGC
SCO5320 promotor R	AGACCGTCATCCGGCCCACCTCT
SCO6029 promotor F	CACCAGCCGTCGTTCATCGTTCC
SCO6029 promotor R	GACACCAGGCCTCCTGGGGTGCG
SCO1189-59bp sense	CTCCCGCCGACCTCACGTGCCGGTTACAAATCGC TCAACCTCTGGTTGCGAAGCGGTGA
SCO1189-59bp antisense	TCACCGCTTCGCAACCAGAGGTTGAGCGATTTGT AACCGGCACGTGAGGTCGGCGGGAG
SCO1489-59bp sense	TGACGCAGCAGAGTAACGCTGCGTAACCTCACA GTGAGTTACCAGCCGCGCGGCCGACA

SCO1489-59bp antisense	TGTCGGCCGCGCGGCTGGTAACTCACTGTGAGG TTACGCAGCGTTACTCTGCTGCGTCA
SCO1489-M1-59bp sense	TGACGCAGCAGAGTAACGCTGCACAGCCTCACA GTGAGTTACCAGCCGCGCGGCCGACA
SCO1489-M1-59bp antisense	TGTCGGCCGCGCGGCTGGTAACTCACTGTGAGG CTGTGCAGCGTTACTCTGCTGCGTCA
SCO1489-M2-59bp sense	TGACGCAGCAGAGTAACGCTGCGTAACCTCACA ACGGGTTACCAGCCGCGCGGCCGACA
SCO1489-M2-59bp antisense	TGTCGGCCGCGCGGCTGGTAACCCGTTGTGAGG TTACGCAGCGTTACTCTGCTGCGTCA
SCO1489-M3-59bp sense	TGACGCAGCAGAACAGCGCTGCGTAACCTCACA GTGAGTTACCAGCCGCGCGGCCGACA
SCO1489-M3-59bp antisense	TGTCGGCCGCGCGGCTGGTAACTCACTGTGAGG TTACGCAGCGCTGTTCTGCTGCGTCA
SCO1568-59bp sense	CTCTGGGTGTGCGTGACTCACCTCACGGGGCGG TCACGTTCCTGCGGGGCGGTGGTCTG
SCO1568-59bp antisense	CAGACCACCGCCCCGCAGGAACGTGACCGCCCC GTGAGGTGAGTCACGCACACCCAGAG
SCO2210-59bp sense	GCCGCGCCACAACGAGCCCGGGTAACACGGGGT TCACCTACGGGCAACGGTCGGGAAAT
SCO2210-59bp antisense	ATTTCCCGACCGTTGCCCGTAGGTGAACCCCGTG TTACCCGGGCTCGTTGTGGCGCGGC
SCO2136-59bp sense	TTCCGCCACAACTCACCTGCAGTGACGTCTTCCT GACACCACGTTGCGTGACGGTTCCG
SCO2136-59bp antisense	CGGAACCGTCACGCAACGTGGTGTCAGGAAGAC GTCACTGCAGGTGAGTTGTGGCGGAA
SCO3485-59bp sense	ATGCCCGTTGTCGACCAGGCGTGTCACCGTATGG TAAACCGGTTATCTGTGACGCGCGG
SCO3485-59bp antisense	CCGCGCGTCACAGATAACCGGTTTACCATACGGT GACACGCCTGGTCGACAACGGGCAT
SCO3561-59bp sense	AATTACTCTCTATTTACTCGCTGTAATCGAACGGA TACTCAGGAGACGGGAACATGTCA
SCO3561-59bp antisense	TGACATGTTCCCGTCTCCTGAGTATCCGTTCGATT ACAGCGAGTAAATAGAGAGTAATT
SCO3863-59bp sense	CGCCCGACTATACACACCGGCCGATACCGATCGG TGACCTGCGTCGGCGGTCAACTGCC
SCO3863-59bp antisense	GGCAGTTGACCGCCGACGCAGGTCACCGATCGG TATCGGCCGGTGTGTATAGTCGGGCG
SCO5583-59bp sense	ACGACGACCGGCCCCGGCCGTTCACCCACGCGT AACACGCACCGTGCCTTCGTCACGGC
SCO5583-59bp antisense	GCCGTGACGAAGGCACGGTGCGTGTTACGCGTG GGTGAACGGCCGGGGCCGGTCGTCGT
SCO6029-a-59bp sense	GATCTGGTGAGTAACAATCCGAGCAATTCGGACA CACTGTCGATCATTCGAAGATCGAA

SCO6029-a-59bp antisense	TTCGATCTTCGAATGATCGACAGTGTGTCCGAAT TGCTCGGATTGTTACTCACCAGATC
SCO6029-aM-59bp sense	GATCTGGTGAACACGAATCCGAGCAATTCGGAC ACACTGTCGATCATTCGAAGATCGAA
SCO6029-aM-59bp antisense	TTCGATCTTCGAATGATCGACAGTGTGTCCGAAT TGCTCGGATTCGTGTTCACCAGATC
SCO6029-b-59bp sense	TCACAGTCGTCTTCGGCACCGAACGCGTCCGCC TTTAGAGAATGATCACGATCTGGTGA
SCO6029-b-59bp antisense	TCACCAGATCGTGATCATTCTCTAAAGGCGGACG CGTTCGGTGCCGAAGACGACTGTGA
SCO6029-bM-59bp sense	TCACAGTCGTCTTCGGCACCGAACGCGTCCGCC TTTAGAGAATGACCGTGATCTGGTGA
SCO6029-bM-59bp antisense	TCACCAGATCACGGTCATTCTCTAAAGGCGGACG CGTTCGGTGCCGAAGACGACTGTGA
SCO7434-59bp sense	ATACCGGTCCGGTGTGTCTGCGTGACGGAGCGG TTACGGACAGCATCATGAGCTCTGCA
SCO7434-59bp antisense	TGCAGAGCTCATGATGCTGTCCGTAACCGCTCCG TCACGCAGACACACCGGACCGGTAT
SCO7458-59bp sense	GGGCGACCGGTGGTCACCTGGTGTAACAGTCTT CTTATGATCTGTAGACGAAATGATCG
SCO7458-59bp antisense	CGATCATTTCGTCTACAGATCATAAGAAGACTGT TACACCAGGTGACCACCGGTCGCCC
**For Real-time PCR analysis**	
SCO1674 realtime F	CGGCACGAGTTCGGGTTC
SCO1674 realtime R	GTTGAGGACGCCGACCAC
SCO1675 realtime F	TCGCCGACTCCGGTGCCCAGGGT
SCO1675 realtime R	AAGGCGGGGTTCAGCAGACCGAT
SCO1800 realtime F	CGTCGCCTCGGGCAACCT
SCO1800 realtime R	ACGCCCAGGTTGCCGAAG
SCO1950 realtime F	CGGTCTGGTGGACGGTCGGG
SCO1950 realtime R	GCCTTGGCCGGGATCGACAG
SCO2136 realtime F	GGCGTCCCACCGTCGTCCCA
SCO2136 realtime R	GGCTTCTCGCTCGGGGCGGC
SCO2699 realtime F	GTGTCCTGTCCGGCAACGTCGTC
SCO2699 realtime R	GCCGTAGCCGCCGCTCTTGTCGT
SCO2705 realtime F	AACGTGGTGCAGGTCCCG
SCO2705 realtime R	TCAGTCGTTCTCGCACTCGTTGC
SCO2716 realtime F	GTCCTCGACTTCGGCCTC
SCO2716 realtime R	TGCCGCAGAGGTTGACCG
SCO2717 realtime F	CGAGGGTGCCGCCGTAGG
SCO2717 realtime R	TTCAGCAGACCAACGAC
SCO2718 realtime F	CTGTGATCGGCATGTCGG

SCO2718 realtime R	AGTTGCCGAACGCCGACT
SCO2719 realtime F	CGATGACCACGGGCAACA
SCO2719 realtime R	GACCGATGACACTGACCG
SCO2792 realtime F	ATGAGCCACGACTCCACC
SCO2792 realtime R	GATCCCGAACACCGACAGC
SCO3034 realtime F	CCCCGAGTCCTTCTTCCCCG
SCO3034 realtime R	GGCGTTCCCTCTCGGACAGG
SCO3323 realtime F	TACCGCTACATCTACTACCG
SCO3323 realtime R	TGGTAGGTGAAGGTGCCGAT
SCO3485 realtime F	GGTGACCAAGTCCGTTGTCT
SCO3485 realtime R	CGGAGTTCGACAGATCAGGG
SCO3549 realtime F	AACGACGGGAGTTTCCACCT
SCO3549 realtime R	GTGAATGGGGAACACCTTGG
SCO3863 realtime F	ACCAGCATCGTCACCGCCTT
SCO3863 realtime R	GCTCTCGCTGTTGCTGTGCG
SCO4091 realtime F	ATGACCGCTCGCACCCCTGA
SCO4091 realtime R	CGGATCGACGTGAGCTTGCC
SCO4543 realtime F	GAGTCAGGTTGAGGCAGGCTT
SCO4543 realtime R	CCTCGGAACTCCTCCCACCA
SCO4767 realtime F	GCTCGCTCTTCTTCCATCCG
SCO4767 realtime R	AGCCCGCCCCAGACGCCGTA
SCO4768 realtime F	CGGAGGACTTCCTCGCCGTT
SCO4768 realtime R	GACTCCCCGCTTGCCCGAGA
SCO5112 realtime F	CGAGATTGAGGGTGCTGA
SCO5112 realtime R	GACGACGAAGGCAGAGAT
SCO5113 realtime F	GAGCATTCTCCGTAACCG
SCO5113 realtime R	CTTGCTGTTGTCCTTGGC
SCO5114 realtime F	ATCAGTGCCTTCACCTTC
SCO5114 realtime R	CGCCAGGAAATGCCAGTA
SCO5320 realtime F	CGTGCGGGTCGTCCTGATGCTGG
SCO5320 realtime R	TGAGGAGCCACTGGGTCGGATTC
SCO5321 realtime F	ACAACCACGGCATCCTCCAGGCG
SCO5321 realtime R	GAGGTTCGTGGCGTGCGTGAGGA
SCO5621 realtime F	GCCGACTTCGTCTCCTCC
SCO5621 realtime R	CCTTCTGCCGCACCGACC
SCO5723 realtime F	GGAACACGAGGAGCGGGTGGAGA
SCO5723 realtime R	CTCCAGGTCGAACTCCCCGTCCC
SCO5819 realtime F	TGATGACCGCCGCCCGTT
SCO5819 realtime R	CCACCCCTTCCCAGGAGG
SCO6029 realtime F	GCAAGCCTGGTCGCCTACCGCCC

SCO6029 realtime R	GTTGCCGATGCGGGGACGAGCCT
SCO6681 realtime F	CAGCGTCGTCTCCGTCAGCAACC
SCO6681 realtime R	GCGGTGCGGGGCGTCGTAGAAGT
SCO6682 realtime F	AGACCCCCAAGGAAGAGG
SCO6682 realtime R	GGTGGTGATGCTCAGGCT
SCO6683 realtime F	CGCCACGGTCGCCGCCTA
SCO6683 realtime R	TCGTGTCCGCTCCCTCCA
SCO6684 realtime F	AGCACCACCACGAGCAGGGC
SCO6684 realtime R	CTGACGAACCAGGTGGAGAT
SCO6685 realtime F	TGGTTCGTATTGCCGTGG
SCO6685 realtime R	CTCTCCGTCCACGACACA
SCO7257 realtime F	GGGAGCCTCGGGCGGTAC
SCO7257 realtime R	GACGCTGTTGCCGCTGAC
SCO7434 realtime F	GACTTCCCCCCTTGCGTTCG
SCO7434 realtime R	TGGAACGGGTCGTATCGGCG
