# Supplementary material for: Deletion of MtrA Inhibits Cellular Development of Streptomyces coelicolor and Alters Expression of Developmental Regulatory Genes
Source: Front Microbiol. 2017 Oct 16;8:2013. doi: 10.3389/fmicb.2017.02013 (PMC5650626; doi:10.3389/fmicb.2017.02013)
Supplement: Supplementary file 3 [file Table_3.PDF]

Table S3. Potential MtrA sites in *Streptomyces coelicolor*

#	Gene	Gene annotation	Potential MtrA site	Score	Position
1	SCO2387	fabD, malonyltransferase	CGTCACCGGGTGGTCAC	5.2	-186
2	SCO2529	putative metalloprotease	AGTGACAGTGCGGTCAC	5.17	-37
3	SCO7434	putative lipoprotein	CGTGACGGAGCGGTTAC	5.17	-27
4	SCO4777	pkaD	CGTCACAGAGCGGTTTC	4.97	-90
5	SCO4907	afsQ1	TGTCACAGAGCTGTCAC	4.86	-129
6	SCO4908	sigQ			-237
7	SCO2465	hrdA	CGTGACCGAGGGGTTAC	4.8	-101
8	SCO2466	hypothetical protein			-101
9	SCO4793	putative secreted protein	GGTAACGGTCCGGTGAC	4.79	-100
10	SCO4794	putative integral membrane protein			-30
11	SCO6275	cpkA	AGTCACGGCGTGGTAAC	4.77	-147
12	SCO6276	putative secreted protein			-195
13	SCO2978	putative secreted protein	GGTCACGACGCGGTCAC	4.73	-128
14	SCO4029	hypothetical protein	CGTGACCGAGCGGTGAC	4.72	-34
15	SCO6614	putative membrane protein	TGTGACCGGCGGGTCAC	4.71	-163
16	SCO0674	xysA	CGTCACCGCCGGGTCAC	4.67	-184
17	SCO0675	hypothetical protein			-25
18	SCO4727	rpsM	CGTCACGGTCGGGTCAT	4.65	-248
19	SCO4896	putative integral membrane protein	TGTCACCGGACGGTGAC	4.65	-112
20	SCO7103	hypothetical protein	GGTCACTGTCCGGTCAA	4.63	-21
21	SCO4587	hypothetical protein	CGTCACCGGGGTGTCAC	4.61	-136
22	SCO4588	hypothetical protein			-16
23	SCO1488	pyrR	ACTCACTGTGAGGTTAC	4.6	-238
24	SCO1489	BldD			-76
25	SCO1189	hypothetical protein	CCTCACGTGCCGGTTAC	4.59	-67
26	SCO1224	putative sugar-phosphate isomerase	CCTCACGTGCCGGTTAC	4.59	-28
27	SCO2672	putative membrane protein	GGTCACAATCCGGTCAA	4.59	-101
28	SCO7125	conserved hypothetical protein	CGTCACGGGGTCGTCTC	4.55	-230
29	SCO7126	hypothetical protein			-66
30	SCO5411	putative integrase/recombinase	CCTTACCGGCCGGTCAC	4.54	-243
31	SCO5413	MarR-transcriptional regulator			-26
32	SCO3116	hypothetical protein	TGTCACGGGAGGGTGAC	4.52	-189
33	SCO3117	hypothetical protein			-121
34	SCO6271	accA1	TCTCACGAGCCGGTAAC	4.52	-221
35	SCO6272	putative secreted protein			-104
36	SCO5732	hypothetical protein	CGTCGCGGGTCGGTTAC	4.5	-160
37	SCO3980	conserved hypothetical protein	CGTCACTCGGCGATCAC	4.49	-28
38	SCO3981	putative transcriptional regulator			-68
39	SCO4779	pkaJ	CGTCACGGGTCTGTGAC	4.49	-151
40	SCO1590	secreted protein	GGTCACGGTTCCGTCTC	4.46	-90
41	SCO1591	3-hydroxyacyl-CoA dehydrogenase			-64

42	SCO4619	putative integral membrane protein	CGTCACACCGCCGTCAC	4.46	-97
43	SCO2301	conserved hypothetical protein	CGTCATCGTTCGGTCAC	4.44	-214
44	SCO2302	hypothetical protein	CGTCATCGTTCGGTCAC		-11
45	SCO6633	putative transcriptional regulator	GGTGACGGTGAGGTAAA	4.43	-151
46	SCO3485	putative transcriptional regulator	TGTCACCGTATGGTAAA	4.42	-105
47	SCO3486	putative aldehyde dehydrogenase			-73
48	SCO1570	argH	CCTCACCGTACGATCAC	4.41	-27
49	SCO1571	hypothetical protein			-109
50	SCO4091	bldC	CGTCACAGAGGCGTGAC	4.41	-247
51	SCO0423	membrane transport protein	CCTCACGGGGCGGCCAC	4.38	-55
52	SCO0424	hypothetical protein			-82
53	SCO1225	hypothetical protein	ACTCACCGGCCGCTCAC	4.38	-130
54	SCO3999	putative lipoprotein	AGTCACCGTGGGTTCAA	4.37	-124
55	SCO7777	putative hydrolase			-104
56	SCO7778	putative transcriptional regulator	CCTCACCGGGTGGGCAC	4.37	-69
57	SCO1444	chitinase precursor	GCTCACAGGGTGGTCAT	4.36	-140
58	SCO4822	putative integral membrane protein	AGTAAGAGTTCGGTCAA	4.36	-139
59	SCO2804	hypothetical protein	TGTGACGGAGCGGCCAC	4.35	-176
60	SCO2805	hypothetical protein			-109
61	SCO3999	putative lipoprotein	TGTGACAGGGCTGTGAC	4.35	-36
62	SCO1275	halogenase	GGTCACCCACCGGTGAC	4.34	-65
63	SCO2461	secreted protein	AGTAACCTGCCGGTTAA	4.34	-179
64	SCO3821	pksC	GGTCACGGAACGGGCAC	4.34	-129
65	SCO4561	putative protein	GATCACAGGCCGGTTAC	4.34	-149
66	SCO6635	pglY	CGTACCCGTCCGGTCAC	4.34	-194
67	SCO6972	hypothetical protein	CGTCCCGGTGCCGTCAC	4.34	-107
68	SCO7657	putative secreted protein	TCTCACAGCGCGGGCAC	4.32	-79
69	SCO3541	putative DNA polymerase	TGTCACGGCTCGGTGTC	4.31	-76
70	SCO5621	whiG	AGTCACGCTACGCTCAC	4.3	-117
71	SCO6385	putative integral membrane protein	AGTCACCCTCGGGTGAC	4.3	-220
72	SCO5028	putative ATP-binding protein	CCCCACCGTGCGGTCAC	4.29	-194
73	SCO3683	putative secreted protein	TGGGACCGGGCGGTCAC	4.28	-56
74	SCO4855	dhsB	AGTCGCGCGGCGGTCAC	4.28	-169
75	SCO1236	ureA	GGTGACGTGGCCGTCAC	4.27	-171
76	SCO7396	putative secreted protein	GGTTACGGTCCCGTAAC	4.27	-97
77	SCO2744	hypothetical protein	GGTCACGATTGGGTCTC	4.26	-201
78	SCO2745	transcriptional regulator			-101
79	SCO5415	icmA,	CGTAACCGGCCCCTCAC	4.26	-98
80	SCO0710	ATP-binding protein	CGTCACCGTGCCGTTCC	4.25	-39
81	SCO2527	hypothetical protein	GGTCACGGAAAGGTCTC	4.25	-101
82	SCO4037	putative small membrane protein	AGTCACCGAGGGTTCAA	4.25	-120
83	SCO5418	putative transcriptional regulator	TGTTACCCGCCGGTTAA	4.25	-26
84	SCO5855	putative DNA-binding protein	GGTAACCGTGCCATAAC	4.25	-152
85	SCO4339	putative reductase	GGTAACCGGATGATCAC	4.24	-53

86	SCO4340	putative integrase			-39
87	SCO0735	oxidoreductase	GCTCACGCTCCGGTTTC	4.23	-172
88	SCO1612	aldehyde dehydrogenase	CGTCACCGACTGGGAAC	4.23	-39
89	SCO3032	putative secreted protein	CCTACCAGGGCGGTCAC	4.23	-223
90	SCO5385	putative dehydrogenase	CGTTACTAACCGGTCAC	4.23	-85
91	SCO7668	conserved hypothetical protein	CCTCACCGAGCGGTTCC	4.22	-202
92	SCO2195	hypothetical protein	GGTCACGCCCTGGTAAC	4.21	-106
93	SCO2201	hypothetical protein	CGTGACACCGCCGTCAC	4.21	-185
94	SCO3006	putative acetyltransferase	TGTGACGGATCGGGCAA	4.21	-236
95	SCO3007	secA			-62
96	SCO3561	putative secreted protein	AGTATCCGTTCGATTAC	4.21	-241
97	SCO3562	putative integral membrane transport protein			-31
98	SCO4129	putative integral membrane protein	AGTGACTGGAAGGTCAC	4.21	-190
99	SCO6230	putative sugar transport system permease protein	CGTCACGGACGGGTCCC	4.21	-124
100	SCO3034	whiB	GGTAACGGCTAGATCAC	4.2	-47
101	SCO5016	putative integral membrane protein	TGTCACCGATCGGGCTC	4.2	-75
102	SCO5017	putative regulatory protein			-149
103	SCO5871	kdpD	CGTCAGGGTTCCGTCAA	4.2	-113
104	SCO3209	putative transcriptional regulator	GGTGACGCGGAGGTGAC	4.19	-75
105	SCO2920	putative secreted protease	GGTCACGATCCGGTGTC	4.18	-196
106	SCO5691	secreted sugar hydrolase	CGTCCCGGCGCGGTAAC	4.18	-54
107	SCO6612	putative hydrolase	GGTCACCCGGAGCTCAC	4.18	-129
108	SCO6630	hypothetical protein	GGCAACAGGTCGGTTAC	4.18	-157
109	SCO1030	hypothetical protein	CGTCACTCTGGGTTTAC	4.16	-165
110	SCO4677	rsfA	AGTCACTCAGCGGGTAC	4.16	-52
111	SCO4678	conserved hypothetical protein			-77
112	SCO7371	hypothetical protein	CCTGACCGGGCGTTAAC	4.16	-85
113	SCO1863	hypothetical protein	CGTCTCGCACGGGTTAC	4.15	-126
114	SCO1864	acetyltransferase			-193
115	SCO2210	glutamine synthetase	GGTAACACGGGGTTCAC	4.15	-141
116	SCO5716	putative peptide transport system secreted peptide binding protein	GGTCACGTATGGGTCAA	4.15	-177
117	SCO1613	glutamine synthetase	CGTCACGCTCCCGGCAC	4.14	-83
118	SCO2887	putative membrane protein	CGTGACCGTCCGGGGAC	4.13	-214
119	SCO6158	hypothetical protein	TGTCACCTGTCCGTCAA	4.13	-121
120	SCO6159	transcriptional regulator			-64
121	SCO1742	ATP-binding protein	GGTCACACGGCCTTCAC	4.12	-127
122	SCO1743	transmembrane protein			-114
123	SCO4904	putative integral membrane protein	GGTCACGGGCGGCTGAC	4.12	-142
124	SCO5276	conserved hypothetical protein	CGAGACCGGCCGGTCAC	4.12	-119
125	SCO5446	probable metalloprotease	GGTCACACGGCCTTCAC	4.12	-182
126	SCO7176	putative secreted peptidase	TGTCACCATGTGGACAC	4.12	-110

127	SCO7177	hypothetical protein			-142
128	SCO1375	hypothetical protein	AGTGACAGTGCGGTTCA	4.11	-214
129	SCO2610	mreC	CGTAACACTTCGGTTCC	4.11	-182
130	SCO4595	putative oxidoreductase	AGTCACGGTGCGCGGAC	4.11	-88
131	SCO5386	putative anti-sigma factor antagonist	GGTATCGGTTCGCTTAC	4.11	-147
132	SCO7095	putative hydrolase	CATAACAGTGTGATTAC	4.11	-69
133	SCO7096	putative membrane protein			-186
134	SCO7112	ECF-family RNA polymerase sigma factor	TGTCACGGATCGATCGC	4.11	-183
135	SCO7113	putative integral membrane protein			-65
136	SCO3439	hypothetical protein	TGTCACCTGGCGGACAC	4.1	-183
137	SCO4941	hypothetical protein	CGGCTCAGTGCGGTCAC	4.1	-38
138	SCO6419	hypothetical protein	CGTCACCGCCCGGCCAC	4.1	-117
139	SCO6768	probable transketolase	CGTCACCGTGAGCTGAA	4.1	-41
140	SCO4885	putative lipoprotein	GCTCACGGGCGGATAAC	4.09	-124
141	SCO6059	hypothetical protein	AGTACCCGTGCGATCAC	4.09	-25
142	SCO6508	gvpK	CCTCACCGTTCGGGGAC	4.09	-21
143	SCO2231	malE	GGTCGCCGAGAGGTCAC	4.08	-193
144	SCO2232	malR			-215
145	SCO6378	putative membrane protein	CGTGACGTGGTGGGTAC	4.08	-110
146	SCO7393	putative lipoprotein	GCTCACCCGCCCGTCAC	4.08	-134
147	SCO7394	putative integral membrane protein			-99
148	SCO0283	hypothetical protein	CGTCAGCGCGCGCTCAC	4.06	-115
149	SCO0284	probable secreted alpha-galactosidase			-69
150	SCO0802	hypothetical protein	CGTCCCGTGTCGGTCAC	4.06	-171
151	SCO1434	CbxX/CfqX family protein	GGTCACCGTCGGGTCGC	4.06	-224
152	SCO4558	putative acetyltransferase	GGTCACGGCGGGATCTC	4.06	-103
153	SCO4884	putative lipoprotein	GTTGACAGGGCGGTAAC	4.06	-137
154	SCO2491	putative oxidoreductase	CGTCACGTTCTCATCAC	4.05	-95
155	SCO2492	putative membrane protein			-105
156	SCO7040	gap2	TGTCACGGGGTGGTCGT	4.05	-244
157	SCO0887	transcriptional regulator	CGAAACACTGCGGTAAC	4.03	-100
158	SCO0888	putative secreted protein			-56
159	SCO5887	redQ	CCTGACCGGGCGCTGAC	4.03	-208
160	SCO5888	redP			-188
161	SCO6676	hypothetical protein	CGTCACCGACAGCTCTC	4.02	-190
162	SCO3703	putative substrate binding protein	CGTCACCGTCCCGGGAC	4.01	-56
163	SCO4508	putative cell division-related protein	GGCAACAGGGCGGTGAC	4.01	-104
164	SCO4509	hypothetical protein			-126
165	SCO5413	Possible transcriptional regulator	TGTGACCGGCCGGTAAG	4.01	-27
166	SCO6802	putative DNA-binding protein	CGTAACACGGGCGTCTC	4.01	-40

167	SCO7161	putative 3-oxoacyl-[acyl-carrier protein] reductase	CGTCCCGGTGCCGTGAC	4.01	-114
168	SCO0760	methyltransferase	CGTGACGGCCGGGGTAC	4	-17
169	SCO1030	hypothetical protein	CGTCACGCTGGTCTCAC	4	-181
170	SCO1517	secreted protein	CGTGACGACCCGCTCAC	4	-180
171	SCO3421	conserved hypothetical protein	CGTAACCGGACGAGTAC	4	-114
172	SCO4833	putative phosphorylmutase	TCCCACCGGGGGGTTAC	4	-22
173	SCO6052	putative membrane protein	AGACACAGCGGGGTCAC	4	-47
174	SCO6317	hypothetical protein	AGGGACGGGGCGGTGAC	4	-87

The prediction has been performed with Regpredict (Novichkov et al., 2010) using the sequences in Figure 7.

REFERENCES

Novichkov, P.S., Rodionov, D.A., Stavrovskaya, E.D., Novichkova, E.S., Kazakov, A.E., Gelfand, M.S., Arkin, A.P., Mironov, A.A., and Dubchak, I. (2010). RegPredict: an integrated system for regulon inference in prokaryotes by comparative genomics approach. *Nucleic Acids Res* 38, W299-307.
