# Supplementary material for: Deletion of MtrA Inhibits Cellular Development of Streptomyces coelicolor and Alters Expression of Developmental Regulatory Genes
Source: Front Microbiol. 2017 Oct 16;8:2013. doi: 10.3389/fmicb.2017.02013 (PMC5650626; doi:10.3389/fmicb.2017.02013)
Supplement: Supplementary file 4 [file Image_1.PDF]

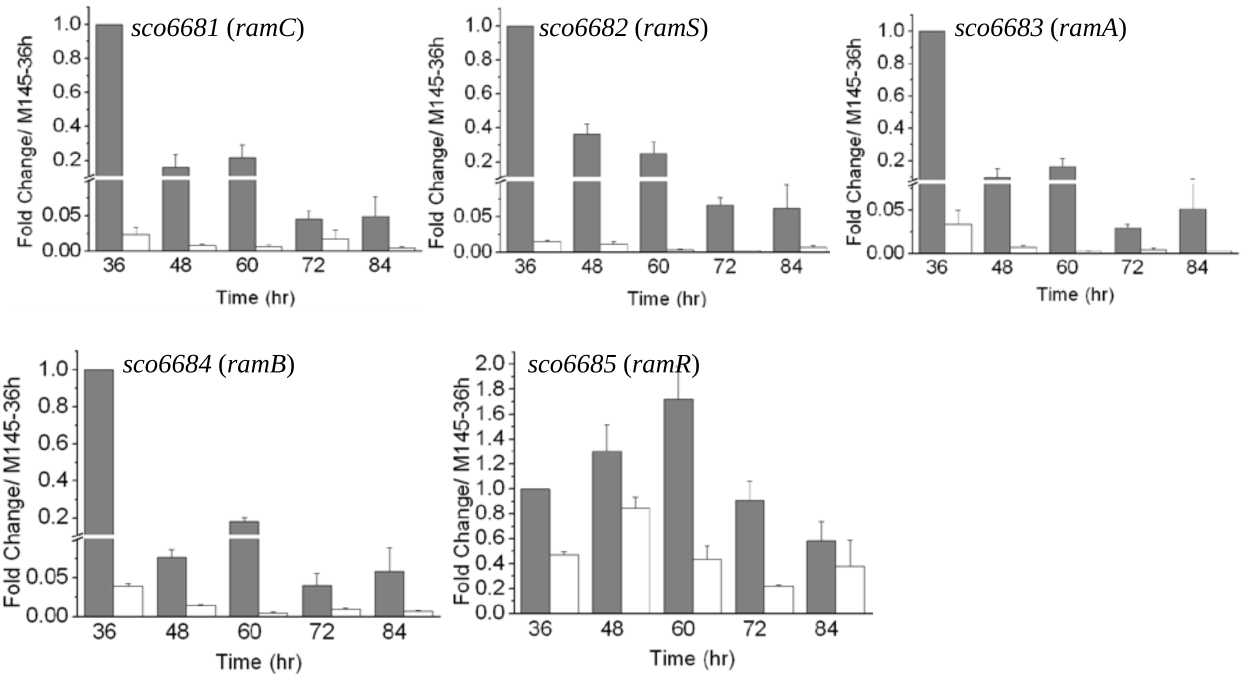

Figure S1. The temporal expression of *ram* genes is dysregulated in the Δ*mtrA* mutant. Gene expression in M145 and Δ*mtrA* was determined by real-time PCR analysis. RNA samples were isolated from M145 and Δ*mtrA* and analyzed as described for Figure 3. The y-axis shows the fold change in expression levels in M145 (grey bars) and Δ*mtrA* (light bars) over the level in M145 at 36 h (arbitrarily set to one). Results are the means (± SD) of triplet experiments.
