# Supplementary material for: Deletion of MtrA Inhibits Cellular Development of Streptomyces coelicolor and Alters Expression of Developmental Regulatory Genes
Source: Front Microbiol. 2017 Oct 16;8:2013. doi: 10.3389/fmicb.2017.02013 (PMC5650626; doi:10.3389/fmicb.2017.02013)
Supplement: Supplementary file 5 [file Image_2.PDF]

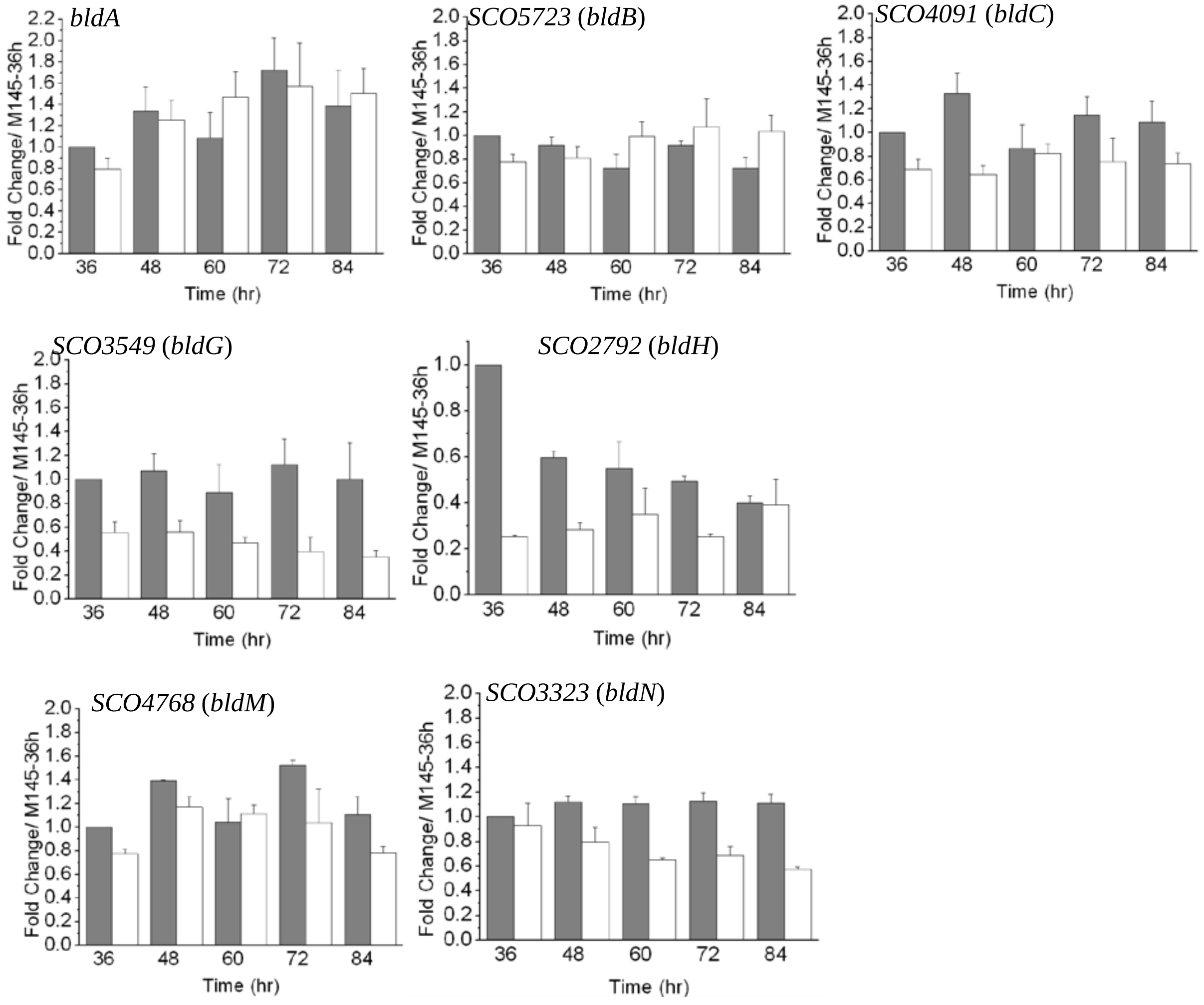

Figure S2. Transcription of *bld* genes in *S. coelicolor* M145 and Δ*mtrA*. RNA samples from M145 and Δ*mtrA* were isolated and analyzed as described for Figure 3. The y-axis shows the fold change in expression levels in M145 (grey bars) and Δ*mtrA* (light bars) over the levels in M145 at 36 h. Results are the means (± SD) of triplet experiments.
