# Supplementary material for: Deletion of MtrA Inhibits Cellular Development of Streptomyces coelicolor and Alters Expression of Developmental Regulatory Genes
Source: Front Microbiol. 2017 Oct 16;8:2013. doi: 10.3389/fmicb.2017.02013 (PMC5650626; doi:10.3389/fmicb.2017.02013)
Supplement: Supplementary file 7 [file Image_4.PDF]

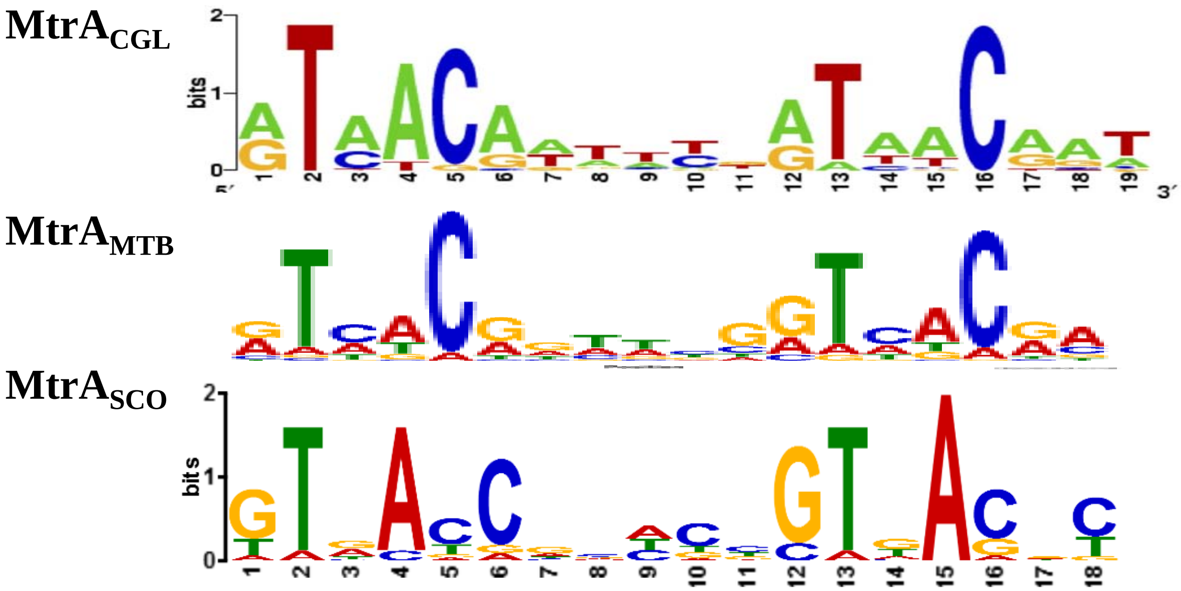

Figure S4. Comparison of the consensus recognition sequencesfor $MtrA_{CGL}$, $MtrA_{MTB}$, and $MtrA_{SCO}$. Consensus sequences for $MtrA_{CGL}$ and $MtrA_{MTB}$ were obtained from previous studies (1, 2).

References

1. **Brocker M, Mack C, Bott M.** 2011. Target genes, consensus binding site, and role of phosphorylation for the response regulator MtrA of Corynebacterium glutamicum. J Bacteriol **193:**1237-1249.

2. **Galagan JE, Minch K, Peterson M, Lyubetskaya A, Azizi E, Sweet L, Gomes A, Rustad T, Dolganov G, Glotova I, Abeel T, Mahwinney C, Kennedy AD, Allard R, Brabant W, Krueger A, Jaini S, Honda B, Yu WH, Hickey MJ, Zucker J, Garay C, Weiner B, Sisk P, Stolte C, Winkler JK, Van de Peer Y, Iazzetti P, Camacho D, Dreyfuss J, Liu Y, Dorhoi A, Mollenkopf HJ, Drogaris P, Lamontagne J, Zhou Y, Piquenot J, Park ST, Raman S, Kaufmann SH, Mohney RP, Chelsky D, Moody DB, Sherman DR, Schoolnik GK.** 2013. The Mycobacterium tuberculosis regulatory network and hypoxia. Nature **499:**178-183.
