# Supplementary material for: Deletion of MtrA Inhibits Cellular Development of Streptomyces coelicolor and Alters Expression of Developmental Regulatory Genes
Source: Front Microbiol. 2017 Oct 16;8:2013. doi: 10.3389/fmicb.2017.02013 (PMC5650626; doi:10.3389/fmicb.2017.02013)
Supplement: Supplementary file 8 [file Image_5.PDF]

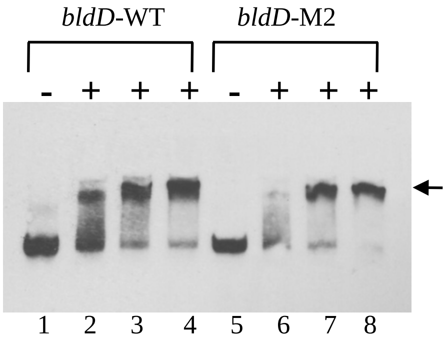

Figure S5. Mutational analyses of putative MtrA binding site in the *bldD* promoter. Mutagenized bases are shown in red in Fig.7C, and the probe (*bldD*-M2) with these mutations was compared with the wild-type sequence for binding to MtrA in EMSAs. Reactions were carried out with the addition of no MtrA (lanes 1, 5); 2.1 μg MtrA (lanes 2, 6); 4.2 μg MtrA (lanes 3, 7); or 6.3 μg MtrA (lanes 4, 8). The arrowhead indicates the positions of the shifted probes.
