# Supplementary material for: Deletion of MtrA Inhibits Cellular Development of Streptomyces coelicolor and Alters Expression of Developmental Regulatory Genes
Source: Front Microbiol. 2017 Oct 16;8:2013. doi: 10.3389/fmicb.2017.02013 (PMC5650626; doi:10.3389/fmicb.2017.02013)
Supplement: Supplementary file 9 [file Image_6.PDF]

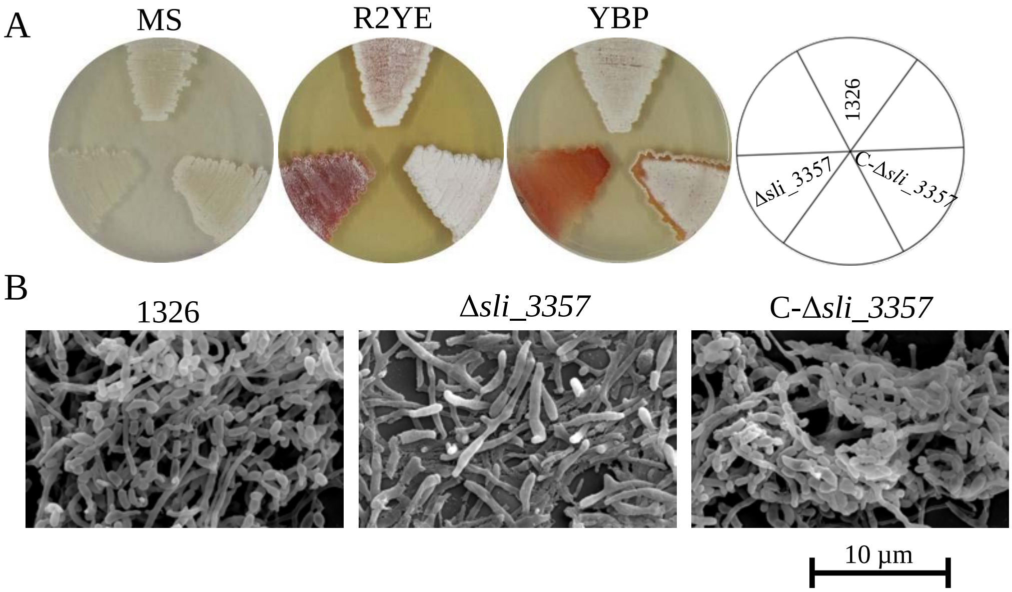

Figure S6. Requirement of the *mtrA* homologous gene*sli_3357* for the formation of aerial hyphae in *S. lividans*. (A) Phenotypes of *S. lividans* strains 1326, Δ*sli_3357*, and C-Δ*sli_3357* grown at 30ºC on solid MS (60 h), R2YE (72 h), and YBP (108 h) media. (B) SEM images of strains 1326, Δ*sli_3357*, and C-Δ*sli_3357* after growth on YBP agar for 96 h. The scale bar is 10 µm.
