# Supplementary material for: Deletion of MtrA Inhibits Cellular Development of Streptomyces coelicolor and Alters Expression of Developmental Regulatory Genes
Source: Front Microbiol. 2017 Oct 16;8:2013. doi: 10.3389/fmicb.2017.02013 (PMC5650626; doi:10.3389/fmicb.2017.02013)
Supplement: Supplementary file 10 [file Image_7.PDF]

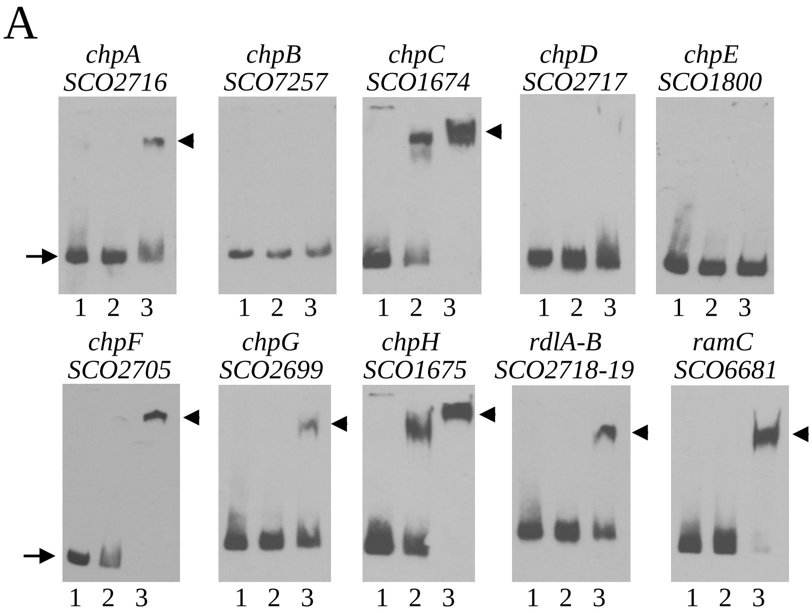

B

ramC	-123	TCGTGTG**GTCACA**CCATC**GCCACG**CCGTTC	-93
rdlA	-243	AAACCCC**GTCCAC**CGGAG**CTCACT**GGTCAA	-213
chpH	-88	GCGCATT**CACTCG**CCCCG**GTCACC**TCCCGC	-118(C)
chpC	-151	TCAGATC**CTCACG**GGGCG**AGGCAC**AACGTC	-181(C)
chpG	-133	CCACGCG**CGGACC**TCGCG**TTCAAA**ACGGGT	-103

Figure S7. Interaction of MtrA with the upstream intergenic sequences of *chp*, *rdl*, and *ram* genes. (A) EMSAs. A fixed amount of labelled probe containing the intergenic region upstream of the indicated gene was incubated in reactions containing no MtrA (lane 1), or 1.8 or 3.6 µg MtrA (lanes 2 and 3, respectively). The DNA fragments used as probes were amplified using specific primer pairs. The length of the probe for each upstream region was as follows: *chpA* (311 bp), *chpB* (253 bp), *chpC* (301 bp), *chpD* (219 bp), *chpE* (267 bp), *chpF* (289 bp), *chpG* (301 bp), *chpH* (293 bp), *rdlA-B* (258 bp), and *ramC* (285 bp). Arrows and arrowheads indicate the positions of the free and the shifted probes, respectively. (B) Predicted MtrA binding sites. The consensus sequences for MtrA recognition are indicated in bold and underlined. Numbers at the right indicate distance from the putative translational start site.
